# Estimated SARS-CoV-2 Seroprevalence in the US as of September 2020

**DOI:** 10.1001/jamainternmed.2020.7976

**Published:** 2020-11-24

**Authors:** Kristina L. Bajema, Ryan E. Wiegand, Kendra Cuffe, Sadhna V. Patel, Ronaldo Iachan, Travis Lim, Adam Lee, Davia Moyse, Fiona P. Havers, Lee Harding, Alicia M. Fry, Aron J. Hall, Kelly Martin, Marjorie Biel, Yangyang Deng, William A. Meyer, Mohit Mathur, Tonja Kyle, Adi V. Gundlapalli, Natalie J. Thornburg, Lyle R. Petersen, Chris Edens

**Affiliations:** 1 COVID-19 Response, US Centers for Disease Control and Prevention, Atlanta, Georgia; 2 ICF Inc, Fairfax, Virginia; 3 Quest Diagnostics, Secaucus, New Jersey; 4 BioReference Laboratories, Elmwood Park, New Jersey

## Abstract

**Importance:**

Case-based surveillance of severe acute respiratory syndrome coronavirus 2 (SARS-CoV-2) infection likely underestimates the true prevalence of infections. Large-scale seroprevalence surveys can better estimate infection across many geographic regions.

**Objective:**

To estimate the prevalence of persons with SARS-CoV-2 antibodies using residual sera from commercial laboratories across the US and assess changes over time.

**Design, Setting, and Participants:**

This repeated, cross-sectional study conducted across all 50 states, the District of Columbia, and Puerto Rico used a convenience sample of residual serum specimens provided by persons of all ages that were originally submitted for routine screening or clinical management from 2 private clinical commercial laboratories. Samples were obtained during 4 collection periods: July 27 to August 13, August 10 to August 27, August 24 to September 10, and September 7 to September 24, 2020.

**Exposures:**

Infection with SARS-CoV-2.

**Main Outcomes and Measures:**

The proportion of persons previously infected with SARS-CoV-2 as measured by the presence of antibodies to SARS-CoV-2 by 1 of 3 chemiluminescent immunoassays. Iterative poststratification was used to adjust seroprevalence estimates to the demographic profile and urbanicity of each jurisdiction. Seroprevalence was estimated by jurisdiction, sex, age group (0-17, 18-49, 50-64, and ≥65 years), and metropolitan/nonmetropolitan status.

**Results:**

Of 177 919 serum samples tested, 103 771 (58.3%) were from women, 26 716 (15.0%) from persons 17 years or younger, 47 513 (26.7%) from persons 65 years or older, and 26 290 (14.8%) from individuals living in nonmetropolitan areas. Jurisdiction-level seroprevalence over 4 collection periods ranged from less than 1% to 23%. In 42 of 49 jurisdictions with sufficient samples to estimate seroprevalence across all periods, fewer than 10% of people had detectable SARS-CoV-2 antibodies. Seroprevalence estimates varied between sexes, across age groups, and between metropolitan/nonmetropolitan areas. Changes from period 1 to 4 were less than 7 percentage points in all jurisdictions and varied across sites.

**Conclusions and Relevance:**

This cross-sectional study found that as of September 2020, most persons in the US did not have serologic evidence of previous SARS-CoV-2 infection, although prevalence varied widely by jurisdiction. Biweekly nationwide testing of commercial clinical laboratory sera can play an important role in helping track the spread of SARS-CoV-2 in the US.

## Key Points

**Question:**

What proportion of persons across 52 US jurisdictions had detectable antibodies against severe acute respiratory syndrome coronavirus 2 (SARS-CoV-2) from July to September 2020?

**Findings:**

In this repeated, cross-sectional study of 177 919 residual clinical specimens, the estimated percentage of persons in a jurisdiction with detectable SARS-CoV-2 antibodies ranged from fewer than 1% to 23%. Over 4 sampling periods in 42 of 49 jurisdictions with calculated estimates, fewer than 10% of people had detectable SARS-CoV-2 antibodies.

**Meaning:**

While SARS-CoV-2 antibody prevalence estimates varied widely across jurisdictions, most people in the US did not have evidence of previous SARS-CoV-2 infection.

## Teaser

This cross-sectional study examines the prevalence of persons with SARS-CoV-2 antibodies across the US and changes from July to September 2020.

## Introduction

The first severe acute respiratory syndrome 2 (SARS-CoV-2) infection in the US was identified in January 2020,^1^ followed soon after by reports of community transmission.^2,3,4,5^ The US remains severely affected by the coronavirus disease 2019 (COVID-19) pandemic, with more than 9 million cases and 230 000 deaths reported through November 1, 2020.^6^ With limited testing availability and mild and asymptomatic infections contributing to underascertainment of SARS-CoV-2 infections through passive case reporting,^7,8,9^ seroprevalence surveys are important for refining estimates of infection and transmission.^10^

Most seroprevalence surveys conducted in the US thus far have been limited to specific geographic areas,^11,12^ focused on unique high-risk populations,^13,14^ or not designed for repeated sampling over time.^15^ Testing of commercial clinical laboratory residual sera has offered a practical, scalable approach to estimate in a more general population the prevalence of persons who develop SARS-CoV-2 antibodies over repeated time intervals.^10,16^

In a nationwide expansion of commercial clinical laboratory serologic testing, we aim to understand how seroprevalence varied across different geographic regions, sexes, age groups, and periods. In this biweekly, repeated cross-sectional study, we tested for SARS-CoV-2 antibodies using sera from persons across the 50 US states, the District of Columbia, and Puerto Rico who sought clinical care. Initial findings from the first testing period were released on the US Centers for Disease Control and Prevention (CDC) website (COVID Data Tracker).^17^ In this article, we present seroprevalence estimates from specimens collected over 4 periods from July to September 2020.

## Methods

### Study Design

Residual patient sera from specimens collected for routine screening (eg, cholesterol, thyroid) or clinical management by 2 commercial laboratories (laboratory A and laboratory B) across 50 US states, Washington DC, and Puerto Rico between July 27 and September 24, 2020, were analyzed. Approximately every 2 weeks, we selected a convenience sample of residual sera from the pool of all available, deduplicated specimens to target equal sample numbers in 4 age groups (0-17 years, 18-49 years, 50-64 years, and ≥65 years) in each jurisdiction. Because laboratory A completed biweekly sampling 3 days after laboratory B for each period, the total number of days included in each period across all jurisdictions was slightly more than 2 weeks. To reduce selection bias, the laboratories reviewed tests that were ordered on the same day as the specimen identified in the convenience sample and excluded the specimen if any requests for SARS-CoV-2 antibody testing were noted.

Laboratory A collected specimens from 7 jurisdictions (Arizona, Indiana, Maryland, Pennsylvania, New Jersey, New York, and Virginia), and laboratory B, which was involved in an earlier CDC-led seroprevalence survey,^10^ provided residual sera from the remaining 45 jurisdictions. Each performed chemiluminescent immunoassay testing for SARS-CoV-2 antibodies and provided CDC with deidentified information on patient age, sex, state, and specimen collection date. The zip code of residence and ordering clinician zip code were also collected. For both laboratories, most specimens were collected in the outpatient setting, although individual-level data on the source of specimens were not available. Information on patient race/ethnicity and symptoms was also not available.

This activity was reviewed by CDC and determined to be consistent with non–human participant research activity.^18^ Informed consent was waived, as data were deidentified. Reporting of this study followed the Strengthening the Reporting of Observational Studies in Epidemiology statement.^19^

### Laboratory Methods

Each laboratory processed and transported specimens according to standard procedures. Most specimens did not require −4 °F storage, and none more than a single thaw cycle. Laboratory A tested all specimens at a central facility using the Roche Elecsys Anti-SARS-CoV-2 pan-immunoglobulin immunoassay that targets the nucleocapsid protein and has a sensitivity of 100% (95% CI, 88.3%-100.0%) and specificity of 99.8% (95% CI, 99.7%-99.9%). Specimens were considered reactive at a cutoff index of 1.0 or greater without serum dilution.^20^ Laboratory B performed testing at 19 regional facilities on samples from 45 jurisdictions using the Abbott ARCHITECT SARS-CoV-2 IgG immunoassay targeting the nucleocapsid protein or Ortho-Clinical Diagnostics VITROS SARS-CoV-2 IgG immunoassay targeting the spike protein. Specimens tested by ARCHITECT were considered reactive at a cutoff index of 1.4 or greater, whereas specimens tested by VITROS were considered reactive at a cutoff index of 1.0 or greater. Using these definitions of reactivity, ARCHITECT had a sensitivity of 100.0% (95% CI, 95.8%-100.0%) and specificity 99.6% (95% CI, 99.0%-99.9%); VITROS had a sensitivity of 90.0% (95% CI, 76.9%-96.0%) and specificity of 100.0% (95% CI, 99.1%-100.0%).^20^ An internal comparative study demonstrated 98.5% qualitative result concordance between the ARCHITECT and VITROS platforms.^21^ For all assays, sensitivity was determined in symptomatic persons with real-time reverse transcriptase polymerase chain reaction–confirmed SARS-CoV-2 infection. All assays were granted Emergency Use Authorization by the US Food and Drug Administration and used according to the Instructions for Use provided by the manufacturers.^20,22^

### Statistical Analysis

Power analyses set a target of 980 samples (245 per age group) to be tested per jurisdiction within each 2-week period. Assuming a baseline seroprevalence of 3%,^10^ this sample size was determined to allow for 70% power to detect a 2% increase in seroprevalence.

For each testing period, we calculated overall seroprevalence estimates by jurisdiction, as well as site-specific age group, sex, and metropolitan status according to 2013 Rural-Urban Continuum Codes (RUCC) classification^23^ for states with sufficient samples to support precise subgroup estimates. We used patient residential zip code data (or the ordering clinician’s zip code if the patient’s zip code was missing) to determine county of residence and assigned metropolitan status based on RUCC codes 1 to 3 and nonmetropolitan status RUCC codes 4 to 9. To produce seroprevalence estimates, the samples in each jurisdiction were weighted to the population using iterative poststratification or raking.^24^ Full details on the weighting procedures are included in the eMethods in the Supplement. Briefly, seroprevalence was calculated as the number of reactive specimens divided by the number of specimens tested. Raking was performed across age, sex, and metropolitan status dimensions to create weights that were adjusted to 2018 American Community Survey 5-year population totals for sex, each age category, and metropolitan status.^25^ For the raking process to converge, probabilistic imputation was performed for patients with missing data on sex, age category, or metropolitan status.

Confidence intervals were calculated using bootstrap resampling.^26^ For each bootstrap resample, false-positive and false-negative rates were generated from a binomial distribution using results from the assay performance specifications.^20^ These rates were applied to the bootstrap resample, raked as described earlier in the article, and the seroprevalence was estimated. The process was repeated 500 times and 95% CIs were calculated from 2.5th and 97.5th quantiles of the bootstrap distribution. We report the final adjusted seroprevalence estimate as the mean of the bootstrap distribution. Estimates based on fewer than 75 specimens were not reported because of potential instability.

Finally, seroprevalence estimates were used to predict the total number of SARS-CoV-2 infections in each jurisdiction by applying the estimated seroprevalence to each site’s population.^25^ To determine the ratio of estimated to reported infections, we assumed that most persons develop detectable antibodies by 7 to 14 days following infection.^27^ We then divided the estimates of total infections per testing period by cumulative reported case counts^28^ as of 14 days before the median collection date for each jurisdiction. SAS Software, version 9.4 (SAS Institute), and R, version 3.6.3 (R Core Team), were used for data management and analyses.

## Results

During 4 collection periods between July 27 and September 24, 2020, we tested 177 919 residual sera specimens from all 50 states, Washington DC, and Puerto Rico (Table). Of all specimens, 103 771 (58.3%) were from women, 26 716 (15.0%) were from persons 17 years or younger, and 47 513 (26.7%) were from persons 65 years or older. The Abbott ARCHITECT and Ortho-Clinical Diagnostics VITROS assays were the most commonly used assays, accounting for 84 815 (47.7%) and 65 258 tests (36.7%), respectively. The remaining 27 846 specimens (15.7%) were tested using the Roche Elecsys assay. Specimens were collected from 2496 of 3141 US counties (79.5%) (eFigure 1 in the Supplement), and 26 290 specimens (14.8%) were from persons residing in nonmetropolitan areas (Table).

**Table.** Demographic and Assay Characteristics of Sampled Populations in 50 US States, Washington DC, and Puerto Rico During 4 Periods of SARS-CoV-2 Testing From July 27 to September 10, 2020

Characteristic	No. (%)^a^			
	Period 1	Period 2	Period 3	Period 4
Total samples	38 776	45 907	45 327	47 909
Dates of specimen collection^b^	July 27-August 13, 2020	August 10-27, 2020	August 24-September 10, 2020	September 7-24, 2020
Sex				
Male	16 024 (41.3)	18 794 (40.9)	18 983 (41.9)	20 343 (42.5)
Female	22 751 (58.7)	27 112 (59.1)	26 344 (58.1)	27 564 (57.5)
Age category, y				
0-17	6700 (17.3)	6920 (15.1)	6484 (14.3)	6612 (13.8)
18-49	11 237 (29.0)	14 571 (31.8)	14 079 (31.1)	15 157 (31.6)
50-64	10 367 (26.8)	12 514 (27.3)	12 426 (27.4)	13 207 (27.6)
≥65	10 408 (26.9)	11 856 (25.9)	12 316 (27.2)	12 933 (27.0)
Assay^c^				
Abbott ARCHITECT	18 467 (47.6)	20 436 (44.5)	22 378 (49.4)	23 534 (49.1)
Ortho VITROS	15 334 (39.6)	17 708 (38.6)	16 116 (35.6)	16 100 (33.6)
Roche Elecsys	4975 (12.8)	7763 (16.9)	6833 (15.1)	8275 (17.3)
Metropolitan status^d^				
Nonmetropolitan	5932 (15.3)	6339 (13.8)	6807 (15.0)	7212 (15.1)
Metropolitan	32 828 (84.7)	39 555 (86.2)	38 500 (85.0)	40 671 (84.9)

^a^ Percentages calculated out of nonmissing values for all periods. Missing values in period 1 included sex (n = 1), age (n = 64), and metropolitan status (n = 16). Missing values in period 2 included sex (n = 1), age (n = 46), and metropolitan status (n = 13). Missing values in period 3 included age (n = 22) and metropolitan status (n = 20). Missing values in period 4 included sex (n = 2) and metropolitan status (n = 26).

^b^ Because laboratories completed biweekly sampling 3 days apart, the total number of days included in each period across all jurisdictions was more than 2 weeks. There were 207 missing dates in period 1, 216 missing dates in period 2, 236 missing dates in period 3, and 226 missing dates in period 4.

^c^ Abbott ARCHITECT SARS-CoV-2 IgG, Ortho-Clinical Diagnostics VITROS Anti-SARS-CoV-2 IgG, and Roche Elecsys Anti-SARS-CoV-2.

^d^ Determined by the 2013 Rural-Urban Continuum Codes classification.^23^ Counties identified by Rural-Urban Continuum Codes 1 to 3 were designated metropolitan and 4 to 9 were designated nonmetropolitan.

**Figure 1.**
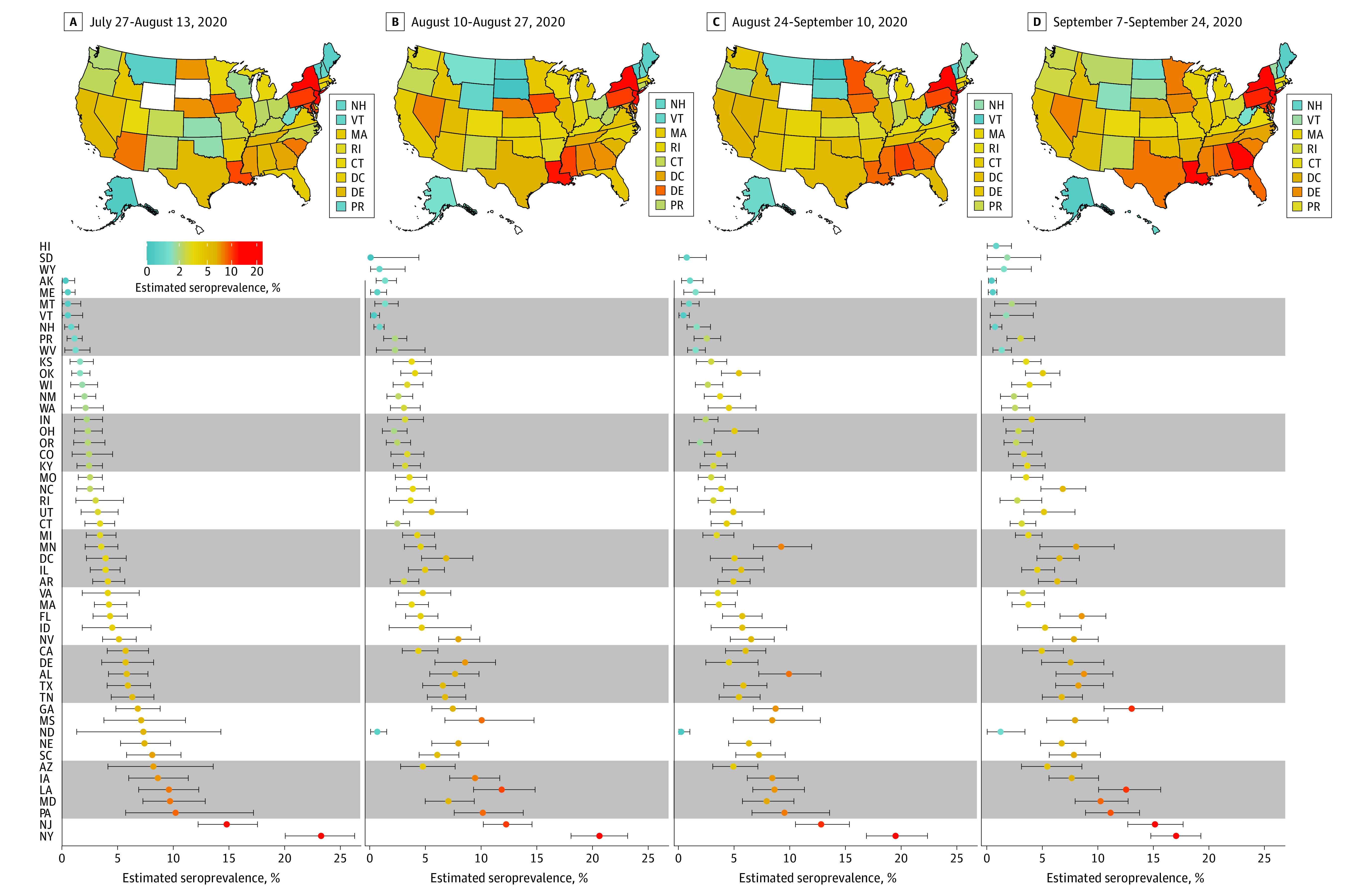
SARS-CoV-2 Prevalence Estimates by US Jurisdiction During Testing Periods From July 27 to August 13, August 10 to 27, August 24 to September 10, and September 7 to 24, 2020 Estimates are shown with 95% bootstrap CIs. Estimates could not be calculated for Hawaii, North Dakota, South Dakota, and Wyoming during select periods during which there were fewer than 75 samples.

Seroprevalence estimates were calculated by jurisdiction over the 4 collection periods (Figure 1 and eTables 3-6 in the Supplement). Seroprevalence ranged from 0.0% (95% bootstrap CI, 0.0%-4.4%) in South Dakota in period 2 to 23.3% (95% bootstrap CI, 20.1%-26.3%) in New York in period 1. In jurisdictions with enough tested samples to calculate an estimate, which included 46 of 49 sites (93.3%) in period 1, 46 of 51 sites (90.2%) in period 2, 48 of 50 sites (96.0%) in period 3, and 46 of 52 sites (88.5%) in period 4, fewer than 10% of specimens had detectable SARS-CoV-2 antibodies.

**Figure 2.**
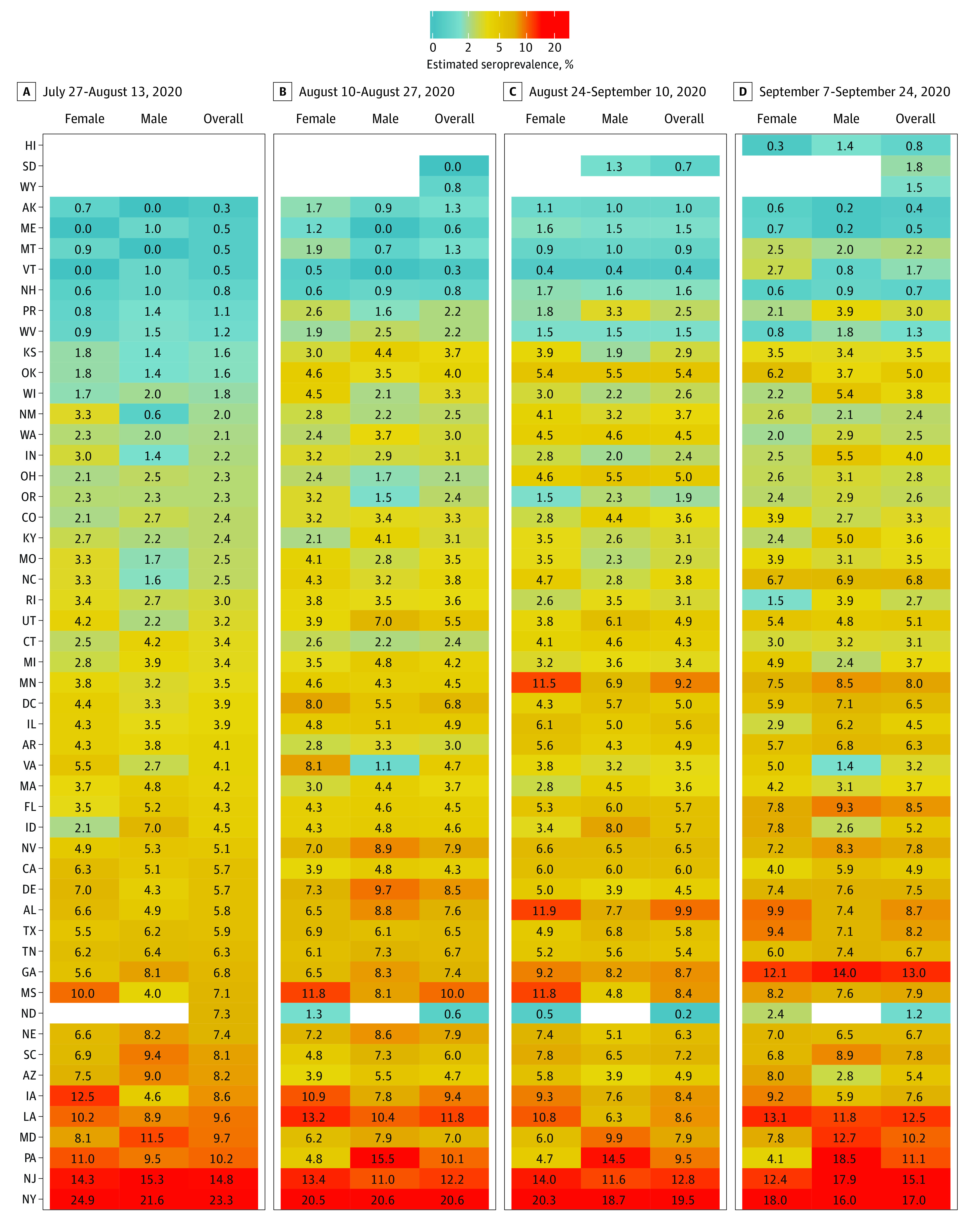
Sex-Stratified SARS-CoV-2 Prevalence Estimates by Jurisdiction During Testing Periods 1 to 4 Estimates could not be calculated for select jurisdictions where there were fewer than 75 samples or no samples were provided for a given reporting subgroup.

**Figure 3.**
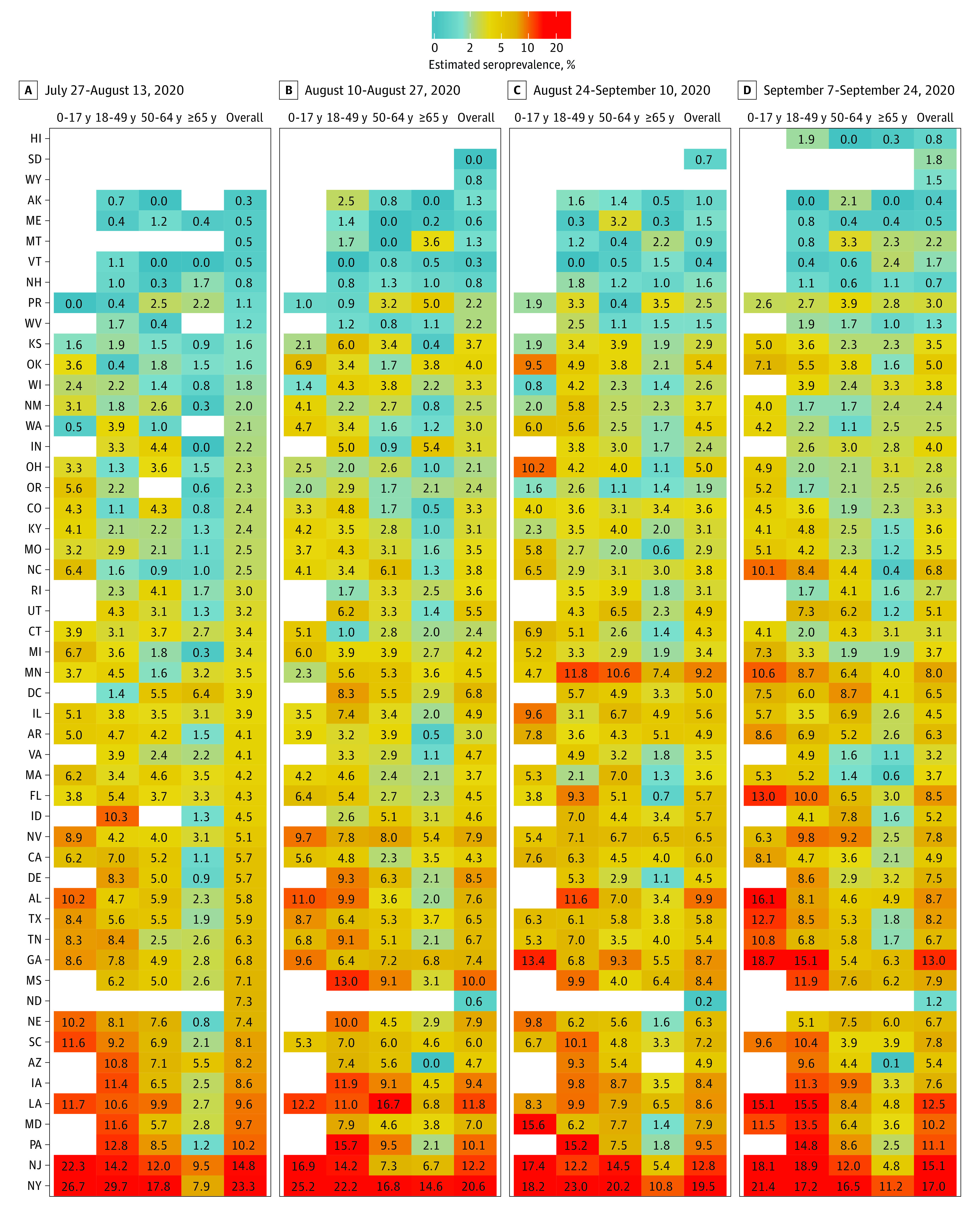
Age-Stratified SARS-CoV-2 Prevalence Estimates by Jurisdiction During Testing Periods 1 to 4 Estimates could not be calculated for select jurisdictions where there were fewer than 75 samples or no samples were provided for a given reporting subgroup.

**Figure 4.**
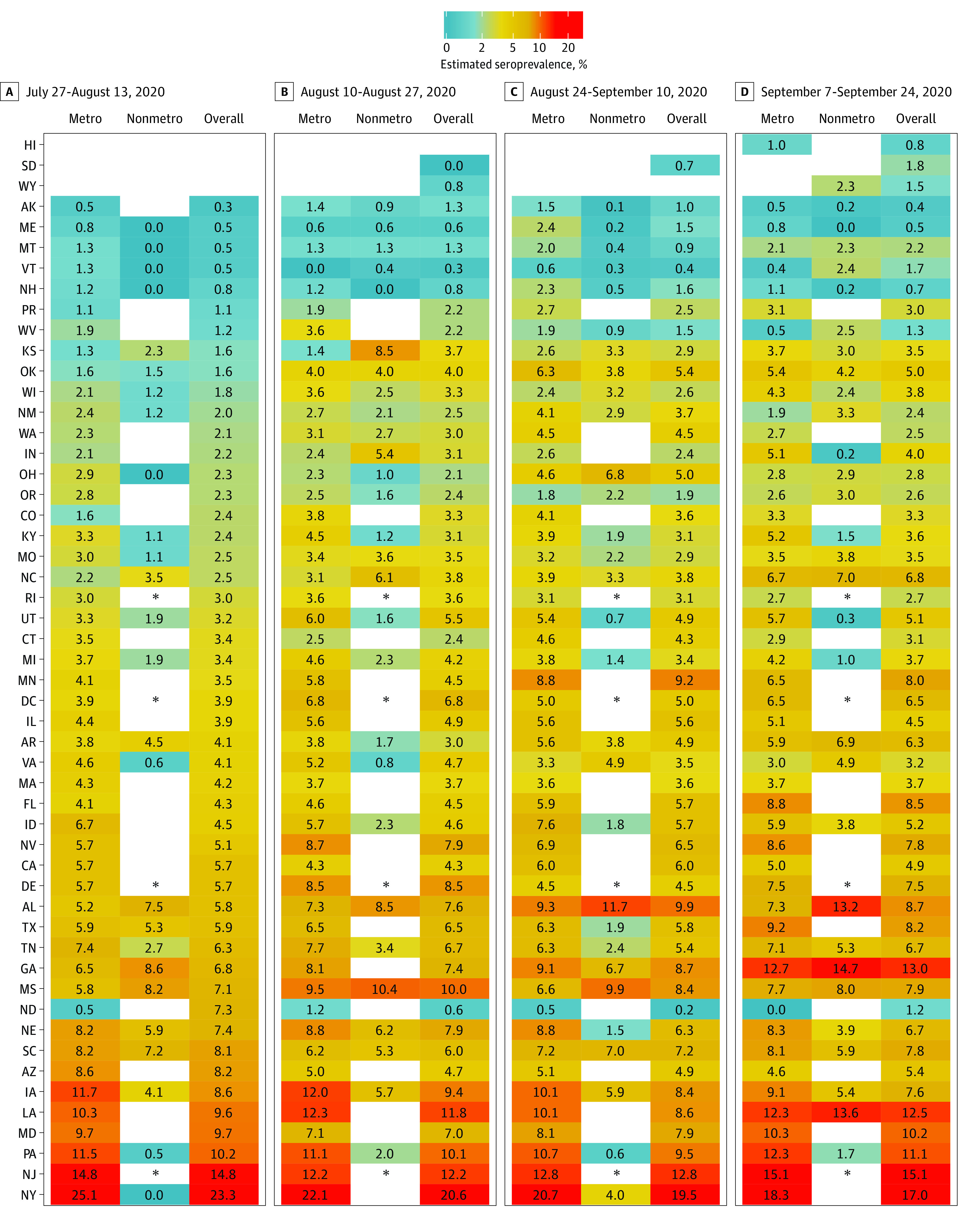
Metropolitan Status–Stratified SARS-CoV-2 Prevalence Estimates by Jurisdiction During Testing Periods 1 to 4 Estimates could not be calculated for select jurisdictions where there were fewer than 75 samples or no samples were provided for a given reporting subgroup. The asterisks indicate that Delaware, New Jersey, Rhode Island, and Washington DC do not contain nonmetropolitan counties.

SARS-CoV-2 prevalence estimates were also calculated by jurisdiction stratified by sex, age group, and metropolitan status during each collection period (Figure 2, Figure 3, and Figure 4 and eTables 3-6 in the Supplement). Overall seroprevalence estimates varied by jurisdiction and period. There were no consistent differences between men and women across all jurisdictions, although in certain states (eg, Iowa, Louisiana, and Mississippi), seroprevalence was higher in women, while in others (eg, Maryland and Pennsylvania) seroprevalence was often higher in men. Seroprevalence in persons 65 years or older was generally lower than in adults age 18 to 49 years. Fewer samples were available for children and adolescents age 0 to 17 years, and among the 26 jurisdictions for which we could estimate seroprevalence across all periods, estimates varied relative to adult age groups. In the 23 jurisdictions with sufficient samples to calculate estimates by metropolitan status, seroprevalence in certain jurisdictions (eg, Iowa, Pennsylvania, and Tennessee) was higher in metropolitan counties, while in others (eg, Alabama and Mississippi) seroprevalence was higher in nonmetropolitan counties.

In 49 jurisdictions with sufficient samples to estimate seroprevalence across all periods, changes from period 1 to 4 varied across sites (Figure 1). The largest absolute percentage point decreases occurred in New York (6.3%) and North Dakota (6.1%), while large increases occurred in Georgia (6.2%) and Minnesota (4.5%). Ratios comparing estimated to reported SARS-CoV-2 infections during periods 1 and 4 ranged from less than 1 in Alaska for both periods to 12.5 in Pennsylvania during period 1 (eTables 1 and 2 in the Supplement).

## Discussion

In a US nationwide survey of SARS-CoV-2 seroprevalence, we tested more than 177 000 residual specimens submitted for non–SARS-CoV-2 testing during 4 periods from July to September 2020 and found that in nearly all jurisdictions, fewer than 10% of people in the US had evidence of previous SARS-CoV-2 infection using currently available commercial IgG assays. Seroprevalence varied across regions and between metropolitan/nonmetropolitan areas, with estimates as high as 23% in the Northeast and 13% in the South, while estimates in the Midwest and West were less than 10%. Seroprevalence was often lowest in older age groups. Changes in seroprevalence over 2 months were generally modest and differed across jurisdictions.

We expanded a previous CDC-led seroprevalence survey from 10 to 52 represented jurisdictions,^10^ broadening the geographic scope and representativeness of SARS-CoV-2 serosurveillance in the US. Early surveys in California and New York focused on distinct community transmission hot spots.^11,12,15^ Others concentrated on high-risk populations, such as health care workers^13,29^ and adult patients receiving dialysis,^14^ in addition to other unique groups like blood donors.^30^ By testing for SARS-CoV-2 antibodies in persons of all ages who are receiving outpatient and inpatient clinical care, we may be better able to estimate seroprevalence in the general US population. Furthermore, our study includes 15% of samples tested from persons living in nonmetropolitan areas, which matches the distribution of US residents^23^ and achieves wider geographic representation than previous nationwide studies.^14^ We also present state-level estimates, whereas other studies were more optimally designed to calculate regional-level estimates.^30^

Our findings add to a growing body of work examining population-level SARS-CoV-2 exposure, as well as differences in transmission across regions. We found that most people in the US did not have evidence of previous SARS-CoV-2 infection. This is consistent with other large-scale seroprevalence surveys conducted in the US,^10,14,30^ as well as population-based surveys in the United Kingdom,^31^ Spain,^32^ and Geneva^33^ that were conducted over periods with substantial SARS-CoV-2 community transmission. Similar to other US surveys, we found the overall prevalence of SARS-CoV-2 to be highest in the Northeast,^10,14,30^ likely reflecting the high incidence of SARS-CoV-2 transmission in New York City (New York) and surrounding regions during the spring and summer of 2020.^34^ While several studies reported higher seroprevalence in more densely populated metropolitan areas,^14,31,32^ our findings were more mixed and reflect the heterogeneity of SARS-CoV-2 transmission across the US.

Similar to numerous other surveys, we found SARS-CoV-2 seroprevalence to be lower in older adults compared with younger adults across nearly all jurisdictions.^14,30,31,32,33^ With endemic coronaviruses, seroprevalence typically increases through childhood into early adulthood,^27^ and a few studies of SARS-CoV-2 have shown seroprevalence to be lower in children and adolescents younger than 18 years compared with young adults.^30,32,33^ Seroprevalence among children and adolescents in our survey was more varied compared with adults and was likely affected by differences in exposure risk across the regions sampled.

The changes in overall seroprevalence over 4 collection periods that spanned 2 months were modest. The 6.1–percentage point reduction in North Dakota was affected by low sample sizes, particularly in nonmetropolitan counties, and is likely not a reflection of true population changes. The few population-level seroprevalence surveys with repeated measurements are generally consistent with small changes over time.^16,30,33^ While the estimates in our study cannot be directly compared with results from an earlier commercial clinical laboratory seroprevalence survey^10,16^ because of differences in the geographic distribution of the 2 study populations, participating laboratories, and SARS-CoV-2 serology tests used in each study, we observed similar patterns of declining seroprevalence in New York and increasing seroprevalence in Minnesota. The ability to conduct repeated commercial clinical laboratory residual sera testing over an extended period will be valuable to assist in the tracking of the jurisdiction-level spread of SARS-CoV-2.

Another potential application of repeated serological surveillance is the calculation of the ratio of estimated to reported SARS-CoV-2 infections. We observed a wide range of ratios across jurisdictions that may be affected by multiple factors, including differences in care-seeking behavior. Therefore, we caution against comparing these ratios across jurisdictions. Instead, monitoring relative changes over time within a jurisdiction may provide a complementary measure of testing capacity and other metrics of public health interest.

Although cross-sectional seroprevalence studies often indicate a higher burden of infection than reported cases alone,^10^ they may still underestimate the total number of prior infections. For one, persons with asymptomatic or mild infection may mount a less robust immune response than persons with more severe disease.^35,36,37,38^ Further, declines in SARS-CoV-2 antibodies following infection have been observed.^35,36,37,38,39^ The kinetics of waning antibodies also appear to differ by type of assay, viral target, and severity of infection^35,40^ We do not yet understand the association of these factors with estimating seroprevalence in the population or interpreting changes in seroprevalence over time.

More research is also needed to fully understand how the presence or absence of SARS-CoV-2 antibodies affects continued susceptibility to the virus and potential immunity in terms of severity of illness once exposed, subsequent recovery, and future reinfection. Large-scale seroprevalence surveys have relied on qualitative immunoassays^10,14,30^ which can be implemented at scale. However, these are not sufficient to estimate correlates of protection against SARS-CoV-2.^41,42^ Furthermore, other elements of innate or cellular immunity may confer protection to SARS-CoV-2 despite the absence of measurable antibodies.^43^ The dynamics of waning antibodies and persistence of B-cell and T-cell memory^44,45,46^ may lead to further underestimation of immunity over time when using qualitative immunoassays. Assays to detect other factors associated with the immune response, such as quantitative antibody levels and neutralizing antibodies for SARS-CoV-2, are resource intensive and not yet widely available.^22^

### Limitations

This cross-sectional study has several important limitations. Persons who have blood taken for routine screening or clinical care may not represent the general US population. They can differ with regard to their underlying health, access to care, care-seeking behavior, exposure risk, or adherence to prevention measures, including use of masks and social distancing.^47^ While we excluded specimens collected for SARS-CoV-2 antibody testing, we could not exclude persons seeking care for COVID-19–related symptoms. The overall direction of bias resulting from these factors is unclear; for example, even if persons with acute SARS-CoV-2 infection were included, they may have presented for care during the window before antibodies could be detected.^27^

The study was not designed to produce a nationwide estimate of seroprevalence, nor does it necessarily represent the demographic or geographic distribution of residents within each jurisdiction. The concordance between the ARCHITECT and VITROS platforms was excellent but did not include comparison with the Roche assay, which further limits the ability to compare estimates across jurisdictions. Information on patient race/ethnicity or important social determinants of health that affect COVID-19 outcomes^14,48,49,50^ was not available, limiting our ability to further refine our weights and adjustments.

The geographic catchment of samples was determined by the distribution of the commercial clinical laboratory testing sites in each jurisdiction, which are often concentrated in urban areas. We also used convenience sampling in selecting from the pool of available commercial specimens, a method which is subject to potential biases. Despite the large size of the study, we did not reach our target sample numbers in all age groups or jurisdictions. We were therefore unable to estimate seroprevalence in Hawaii, South Dakota, and Wyoming for all periods or in the 0- to 17-year age group for many jurisdictions. Low sample numbers in nonmetropolitan counties also limited reliable metropolitan/nonmetropolitan subgroup estimates in several jurisdictions. Finally, specimens were tested using 1 of 3 immunoassays, each with slightly different performance characteristics. The specificity of all 3 assays was 99.6% or greater, while there was a broader range of sensitivity. Although we adjusted for assay performance specifications, including uncertainty based on validation testing, assay sensitivity among symptomatic persons with reverse-transcriptase polymerase chain reaction–confirmed SARS-CoV-2 infection as described in the manufacturer Instructions for Use is expected to be higher than in our study population, in which persons experiencing previous infection may have had milder disease or had blood drawn for antibody testing at differing times since infection.

## Conclusions

In this US nationwide seroprevalence cross-sectional study, we found that as of September 2020, most persons in the US did not have detectable SARS-CoV-2 antibodies, and seroprevalence estimates varied widely by jurisdiction. Continued biweekly testing of sera collected by commercial laboratories will allow for assessment of the changing epidemiology of SARS-CoV-2 in the US in the coming months. Our results reinforce the need for continued public health preventive measures, including the use of face masks and social distancing, to limit the spread of SARS-CoV-2 in the US.

## Notes

Supplement. eMethods. eFigure 1. Geographic distribution of serology specimens by county across 50 US states, Washington D.C., and Puerto Rico eFigure 2. Dates of sample collection for SARS-CoV-2 antibody testing in periods 1-4 eTable 1. Estimated number of infections based on period 1 seroprevalence estimates compared with the cumulative number of reported cases in each jurisdiction eTable 2. Estimated number of infections based on period 4 seroprevalence estimates compared with the cumulative number of reported cases in each jurisdiction eTable 3. Overall, sex-, age-, and metropolitan/non-metropolitan-stratified SARS-CoV-2 prevalence estimates and 95% confidence intervals by jurisdiction during testing period 1 eTable 4. Overall, sex-, age-, and metropolitan/non-metropolitan-stratified SARS-CoV-2 prevalence estimates and 95% confidence intervals by jurisdiction during testing period 2 eTable 5. Overall, sex-, age-, and metropolitan/non-metropolitan-stratified SARS-CoV-2 prevalence estimates and 95% confidence intervals by jurisdiction during testing period 3 eTable 6. Overall, sex-, age-, and metropolitan/non-metropolitan-stratified SARS-CoV-2 prevalence estimates and 95% confidence intervals by jurisdiction during testing period 4
