# Supplementary material for: Estimated SARS-CoV-2 Seroprevalence in the US as of September 2020
Source: JAMA Intern Med. 2020 Nov 24;181(4):450–60. doi: 10.1001/jamainternmed.2020.7976 (PMC7686880; doi:10.1001/jamainternmed.2020.7976)
Supplement: Supplement. — eMethods. eFigure 1. Geographic distribution of serology specimens by county across 50 US states, Washington D.C., and Puerto Rico eFigure 2. Dates of sample collection for SARS-CoV-2 antibody testing in periods 1-4 eTable 1. Estimated number of infections based on period 1 seroprevalence estimates compared with the cumulative number of reported cases in each jurisdiction eTable 2. Estimated number of infections based on period 4 seroprevalence estimates compared with the cumulative number of reported cases in each jurisdiction eTable 3. Overall, sex-, age-, and metropolitan/non-metropolitan-stratified SARS-CoV-2 prevalence estimates and 95% confidence intervals by jurisdiction during testing period 1 eTable 4. Overall, sex-, age-, and metropolitan/non-metropolitan-stratified SARS-CoV-2 prevalence estimates and 95% confidence intervals by jurisdiction during testing period 2 eTable 5. Overall, sex-, age-, and metropolitan/non-metropolitan-stratified SARS-CoV-2 prevalence estimates and 95% confidence intervals by jurisdiction during testing period 3 eTable 6. Overall, sex-, age-, and metropolitan/non-metropolitan-stratified SARS-CoV-2 prevalence estimates and 95% confidence intervals by jurisdiction during testing period 4 [file jamainternmed-e207976-s001.pdf]

Supplemental Online Content

Bajema KL, Wiegand RE, Cuffe K, et al. Estimated SARS-CoV-2 seroprevalence in the US as of September 2020. *JAMA Intern Med.* Published online November 24, 2020. doi:10.1001/jamainternmed.2020.7976

eMethods.

eFigure 1. Geographic distribution of serology specimens by county across 50 US states, Washington D.C., and Puerto Rico, July to September 2020

eFigure 2. Dates of sample collection for SARS-CoV-2 antibody testing in periods 1-4

eTable 1. Estimated number of infections based on period 1 seroprevalence estimates compared with the cumulative number of reported cases in each jurisdiction

eTable 2. Estimated number of infections based on period 4 seroprevalence estimates compared with the cumulative number of reported cases in each jurisdiction

eTable 3. Overall, sex-, age-, and metropolitan/nonmetropolitan-stratified SARS-CoV-2 prevalence estimates and 95% confidence intervals by jurisdiction during period 1

eTable 4. Overall, sex-, age-, and metropolitan/nonmetropolitan-stratified SARS-CoV-2 prevalence estimates and 95% confidence intervals by jurisdiction during period 2

eTable 5. Overall, sex-, age-, and metropolitan/nonmetropolitan-stratified SARS-CoV-2 prevalence estimates and 95% confidence intervals by jurisdiction during period 3

eTable 6. Overall, sex-, age-, and metropolitan/nonmetropolitan-stratified SARS-CoV-2 prevalence estimates and 95% confidence intervals by jurisdiction during period 4

This supplemental material has been provided by the authors to give readers additional information about their work.

© 2020 Bajema KL et al. *JAMA Internal Medicine.*

eMethods

Introduction

This section details our analytic approach to the biweekly computation of weighted seroprevalence estimates for each jurisdiction accompanied by 95% confidence intervals. Our approach accounted for the variance due to sampling, weighting, and measurement error (sensitivity and specificity of the assay tests) and is based on an iterative poststratification process, also known as raking, that capitalizes on the American Community Survey (ACS) population data for each jurisdiction.[1]

Weighting Process

Traditionally, probability sample surveys used to generate population estimates provide a probability of selection for each unit selected from the target population. These probabilities of selection are used to derive sampling weights to make representative population estimates. In this study, samples were generated through non-probability sampling (i.e., convenience sampling). Therefore, proper probabilities of selection could not be calculated.

There has been extensive recent research in the relative advantages and disadvantages of non-probability sampling. A comprehensive review is provided in an AAPOR Task Force Report.[2] This research has included alternative weighting approaches for non-probability samples ranging from simple post-stratification adjustments to more complex propensity matching.[3-5]

In more recent comparisons, an iterative post-stratification method, or raking, has been recommended as an effective standard method.[6-8] This approach is especially recommended when there is no parallel probability sample that can be used to calibrate the non-probability sample on many population dimensions and totals, commonly referred to as control totals. The raking approach allows the use of any number of population dimensions (e.g., demographic variable) without concern for small cell sizes associated with cross classification of multiple variables or variables with large numbers of levels (e.g., age categories × sex × race × income).

Raking Overview

The starting weights (W_{jh}^0) are post-stratified by a factor F_{jh} for each respondent (j) within each level/cell (h) (e.g., age: 0-17) of a single demographic control total.

The adjustment factor is defined as:

$$F_{jh} = \frac{T_h}{\sum W_{jh}^0}$$

Where T_h is the population total within poststratification level/cell h.

The post-stratified weight is then calculated as the product of the starting weight (W_{jh}^0) and the poststratification factor within a level/cell (F_{jh}).

$$W_{jh}^1 = W_{jh}^0 \times F_{jh}$$

This process is repeated for each weighting dimension.

This process can be generalized as:

$$F_{jh}^{i} = \frac{T_h}{\sum W_{jh}^{i}}$$

$$W_{jh}^{i+1} = W_{jh}^{i} \times F_{jh}^{i}$$

Where, *i* signifies the current values of the raking procedure and *i+1* signifies the values to be used in the next iteration.

As the post-stratification factor (*F*) for each demographic dimension is applied to the weights, the weighted totals for the previous demographic dimension will deviate from the population totals. However, as this post-stratification procedure is repeated, these deviations will tend to reduce to zero. Over many iterations, all the weighted demographic dimensions will match the demographic population totals (T).

This adjustment process is repeated until, $F_{jh}^{i} \approx 1$, signifying that the weights have converged and that the weighted sums will equal the population totals for all post-stratification dimensions.

Raking Process

We utilized the 2018 ACS 5-year population totals for age category and sex and 2013 Rural-Urban Continuum Codes for metropolitan and nonmetropolitan categories as raking dimensions. In order to avoid weighting cells that were too sparse or empty for many jurisdictions, we developed an approach to collapse cells which is described in the next section. The resulting weights adjusted for potential bias across age, sex, and metropolitan/nonmetropolitan categories.

For the raking process to converge in each raking stage, all data had to be non-missing for each raking dimension. If data were missing in proposed dimensions, we applied a combination of probabilistic imputation methods in order to arrive at a complete dataset.

Cell Collapsing for Weighting

In the following tables, we document the logical collapsing criteria used to ensure that each weighting cell had at least 2 observations prior to raking. The dark outlines correspond with the groups to be collapsed, which is also described in the "Collapsing Action" column.

For the age raking dimension, to maintain logical groupings across the age dimension, age groupings were collapsed upwards. The distinction between the <18 age group and other age groups was kept where possible.

<18	18-49	50-64	≥65	Collapsing Action
Filled	Filled	Filled	Filled	No collapsing
**Sparse**	Filled	Filled	Filled	Collapse <18 and 18-49
Filled	**Sparse**	Filled	Filled	Collapse 18-49 and 50-64
Filled	Filled	**Sparse**	Filled	Collapse 50-64 and ≥65
Filled	Filled	Filled	**Sparse**	Collapse 50-64 and ≥65
Filled	Filled	**Sparse**	**Sparse**	Collapse 18-49, 50-64, and ≥65

© 2020 Bajema KL et al. *JAMA Internal Medicine.*

Filled	**Sparse**	Filled	**Sparse**	Collapse 18-49, 50-64, and ≥65
Filled	**Sparse**	**Sparse**	Filled	Collapse 18-49, 50-64, and ≥65
**Sparse**	Filled	**Sparse**	Filled	Collapse <18 and 18-49; Collapse 18-49 and 50-64
**Sparse**	Filled	Filled	**Sparse**	Collapse <18 and 18-49; Collapse 18-49 and 50-64
**Sparse**	**Sparse**	Filled	Filled	Collapse <18, 18-49, and 50-64
Filled	**Sparse**	**Sparse**	**Sparse**	Collapse all age groups
**Sparse**	Filled	**Sparse**	**Sparse**	Collapse all age groups
**Sparse**	**Sparse**	Filled	**Sparse**	Collapse all age groups
**Sparse**	**Sparse**	**Sparse**	Filled	Collapse all age groups

For the sex raking dimension, if any cells were sparse both groups were collapsed.

Male	Female	Collapsing Action
Filled	Filled	No collapsing
Filled	**Sparse**	Collapse all sex groups
**Sparse**	Filled	Collapse all sex groups

For the metro raking dimension, if any cells were sparse both groups were collapsed.

Non-Metro	Metro	Collapsing Action
Filled	Filled	No collapsing
Filled	**Sparse**	Collapse all metro groups
**Sparse**	Filled	Collapse all metro groups

Overview of Analysis Process

We extended a previously used estimation approach[9] in several ways, primarily to account for the sampling variability, and used weighted estimates adjusted to the population. In order to account for the variability associated with the measurement error (sensitivity and specificity), this adjustment process was performed within each bootstrap sample.

Measurement Error Adjustment

Each assay has a different sensitivity and specificity as defined by the manufacturer's validation testing platform. The bootstrap loop performed a different adjustment for each type of antibody assay as outlined below:

1. Generate binomial probability distribution using sensitivity and specificity parameters provided by the lab as input;
2. By each age and sex group:
    a. Calculate unadjusted prevalence rates

© 2020 Bajema KL et al. *JAMA Internal Medicine.*

b. Using the sensitivity, specificity, and unadjusted prevalence rates, calculate a false positive rate and false negative rate.
c. Flip at random a proportion of positive and negatives cases corresponding to false positive and false negative rates.

Weighting

Our approach integrated the weighting into the bootstrap replications. With this approach, the raking process took place for each replicated bootstrap subsample together with the adjustments for sensitivity and specificity. In this way, the bootstrap replications accounted for all variability which was reflected in the empirical confidence intervals. This approach followed a similar bootstrapping variance estimation technique by Shao and Sitter's method for variance estimation of imputed survey data.[10]

Bootstrap Estimation

Through bootstrapping, we produced an adjusted prevalence estimate that incorporated both weighting and measurement error adjustments for 500 replicates. Then, drawing from the bootstrap distribution of all replicates, we calculated the point estimate from the mean and 95% confidence intervals from the 2.5th and 97.5th percentiles.

Number of Bootstrap Replications

We sought to empirically identify the ideal replicate sample size for the bootstrap estimation. Simulations were run to determine the optimal number of replications. Our simulation framework was to run 2,000 replicates and then draw subsamples of replicates with replacement for various replicate sizes, from 10 to 1,500.

Estimates were calculated for 49 jurisdictions with enough data at baseline. We calculated the standard error of the measurement adjusted jurisdiction total prevalence estimates at each replicate size, along with a comparison of each standard error to the standard errors of the full 2,000 replicate sample (assumed to be the "best" due to the largest sample size), which was defined as our benchmark.

On average, the largest deviance values across jurisdictions were from the 10-100 replicate size estimates. Decreases to the average standard error across jurisdictions was minimal after 400 replicates (see the following figure). Therefore, we chose 500 replicates.

© 2020 Bajema KL et al. *JAMA Internal Medicine.*

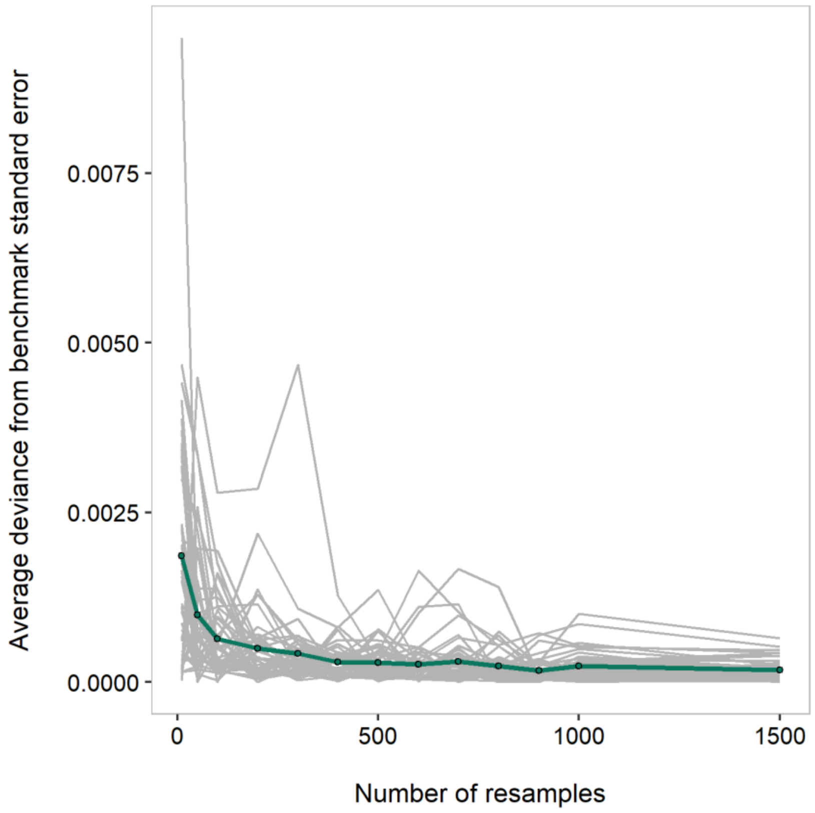

© 2020 Bajema KL et al. *JAMA Internal Medicine.*

Supplemental Figures

eFigure 1. Geographic distribution of serology specimens by county across 50 U.S. states, Washington D.C. and Puerto Rico, July to September 2020

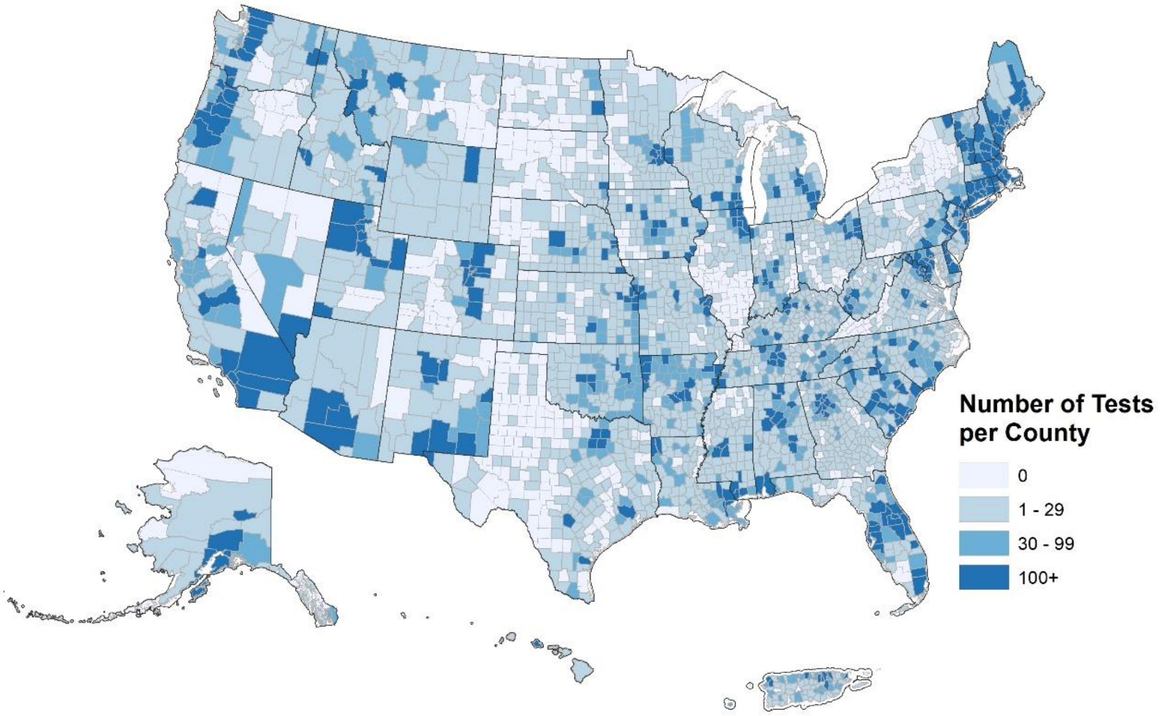

© 2020 Bajema KL et al. *JAMA Internal Medicine.*

eFigure 2. Dates of sample collection for SARS-CoV-2 antibody testing in periods 1-4[a]

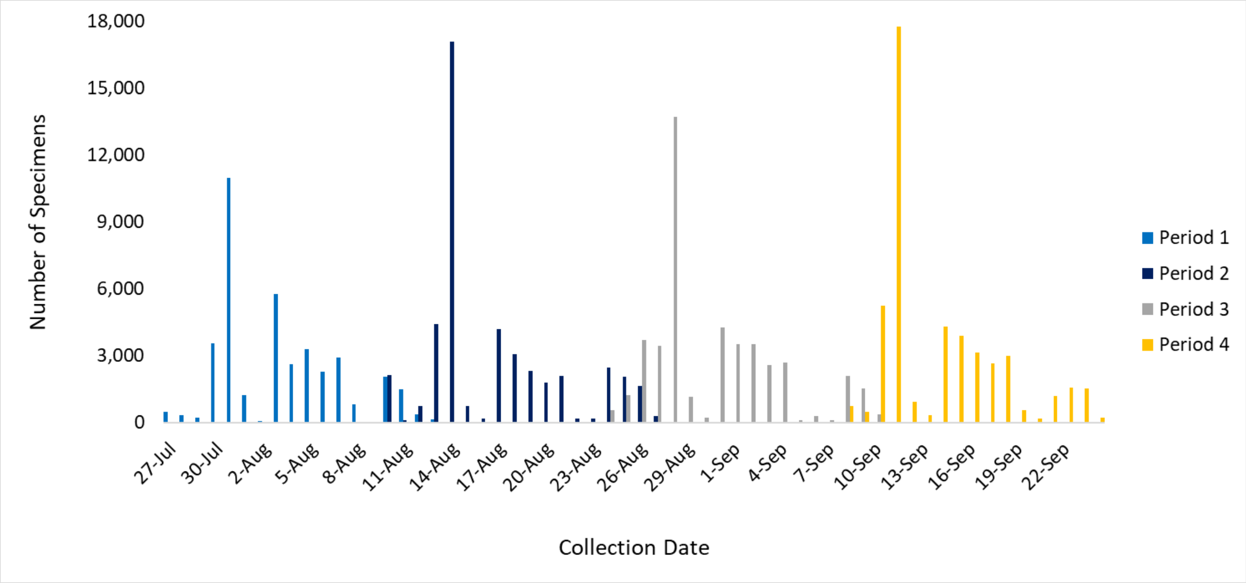

[a]Period 1, July 27 to August 13, 2020; period 2, August 10 to 17, 2020; period 3, August 24 to September 10, 2020; period 4, September 8 to 24, 2020

© 2020 Bajema KL et al. *JAMA Internal Medicine.*

Supplemental Tables

eTable 1. Estimated number of infections based on period 1 seroprevalence estimates compared with the cumulative number of reported cases in each jurisdiction

Jurisdiction	Reported Cases[a]	Estimated Infections[b]	Estimated Infections, Lower[b]	Estimated Infections, Upper[b]	Median Collection Date[c]	Ratio of Estimated Infections to Reported Cases	Ratio Range
AK	2,622	2,216	0	8,271	10-Aug	0.8	0.0-3.2
AL	70,357	282,151	202,371	375,067	4-Aug	4.0	2.9-5.3
AR	34,655	122,618	81,944	168,375	4-Aug	3.5	2.4-4.9
AZ	152,944	569,628	285,509	944,054	6-Aug	3.7	1.9-6.2
CA	370,115	2,231,479	1,585,525	3,041,859	31-Jul	6.0	4.3-8.2
CO	39,344	132,747	49,227	250,561	31-Jul	3.4	1.3-6.4
CT	47,891	121,771	73,063	169,047	31-Jul	2.5	1.5-3.5
DC	11,649	26,695	14,922	39,427	6-Aug	2.3	1.3-3.4
DE	13,624	54,121	33,707	78,048	3-Aug	4.0	2.5-5.7
FL	360,394	885,720	570,568	1,207,051	3-Aug	2.5	1.6-3.3
GA	152,031	700,229	497,368	907,208	5-Aug	4.6	3.3-6.0
HI[d]	1,488	.	.	.	6-Aug	.	.
IA	39,792	269,395	187,323	355,225	5-Aug	6.8	4.7-8.9
ID	18,694	75,951	30,381	134,856	10-Aug	4.1	1.6-7.2
IL	159,333	500,038	323,102	668,000	31-Jul	3.1	2.0-4.2
IN	59,600	146,023	73,012	240,939	6-Aug	2.5	1.2-4.0
KS	21,960	46,540	20,361	81,446	31-Jul	2.1	0.9-3.7
KY	23,414	106,565	58,611	160,735	3-Aug	4.6	2.5-6.9
LA	88,588	447,707	320,857	573,158	31-Jul	5.1	3.6-6.5
MA	112,879	286,868	197,393	396,834	31-Jul	2.5	1.7-3.5
MD	80,172	582,333	435,849	772,642	5-Aug	7.3	5.4-9.6
ME	3,723	6,664	0	15,461	5-Aug	1.8	0.0-4.2
MI	82,392	338,555	215,082	481,942	3-Aug	4.1	2.6-5.8
MN	48,679	193,458	113,864	276,921	6-Aug	4.0	2.3-5.7
MO	31,281	152,252	87,697	219,851	31-Jul	4.9	2.8-7.0
MS	47,071	212,202	112,079	331,454	5-Aug	4.5	2.4-7.0
MT	2,910	5,209	0	17,397	6-Aug	1.8	0.0-6.0
NC	95,478	253,891	133,039	377,789	31-Jul	2.7	1.4-4.0
ND	5,206	54,911	9,779	107,414	5-Aug	10.5	1.9-20.6
NE	22,717	140,952	100,000	185,714	3-Aug	6.2	4.4-8.2
NH	6,164	10,749	2,956	19,886	31-Jul	1.7	0.5-3.2
NJ	177,591	1,314,513	1,086,250	1,560,540	5-Aug	7.4	6.1-8.8
NM	16,456	41,849	22,808	63,192	31-Jul	2.5	1.4-3.8

© 2020 Bajema KL et al. *JAMA Internal Medicine.*

NV	33,288	149,065	106,099	194,662	31-Jul	4.5	3.2-5.8
NY	408,945	4,571,100	3,937,424	5,163,577	5-Aug	11.2	9.6-12.6
OH	72,280	267,763	129,225	420,272	31-Jul	3.7	1.8-5.8
OK	24,139	62,690	33,304	97,562	31-Jul	2.6	1.4-4.0
OR	16,101	93,885	42,044	156,747	7-Aug	5.8	2.6-9.7
PA	104,358	1,304,700	730,376	2,201,362	6-Aug	12.5	7.0-21.1
PR[e]	.	37,256	14,564	60,965	.	.	.
RI	17,291	31,698	12,996	58,219	5-Aug	1.8	0.8-3.4
SC	78,298	401,430	286,948	529,788	7-Aug	5.1	3.7-6.8
SD[d]	8,075	.	.	.	5-Aug	.	.
TN	84,508	419,019	293,313	548,715	5-Aug	5.0	3.5-6.5
TX	311,606	1,645,227	1,126,562	2,216,873	31-Jul	5.3	3.6-7.1
UT	35,582	97,451	51,771	153,181	5-Aug	2.7	1.5-4.3
VA	81,229	344,965	151,448	583,075	6-Aug	4.2	1.9-7.2
VT	1,366	3,125	0	11,500	5-Aug	2.3	0.0-8.4
WA	50,006	153,181	59,084	269,890	6-Aug	3.1	1.2-5.4
WI	45,897	104,011	43,916	183,753	6-Aug	2.3	1.0-4.0
WV	5,548	21,949	4,207	45,726	6-Aug	4.0	0.8-8.2
WY[d]	2,287	.	.	.	5-Aug	.	.

[a]Cumulative cases reported on USAFacts[11] as of 14 days before the median collection date of commercial laboratory residual sera specimens in each jurisdiction.
[b]Estimated infections are calculated by multiplying the overall seroprevalence estimate in each jurisdiction by the underlying population using 2018 American Community Survey 5-year population totals.[1] Lower and upper estimated infections are calculated by multiplying the respective 95% confidence intervals for seroprevalence estimates by the underlying population.
[c]Median collection date of all samples in the corresponding jurisdiction during period 1.
[d]Unable to estimate overall seroprevalence in Hawaii, South Dakota, and Wyoming due to low sample sizes.
[e]Cumulative cases not reported on USAFacts.[11]

© 2020 Bajema KL et al. *JAMA Internal Medicine.*

eTable 2. Estimated number of infections based on period 4 seroprevalence estimates compared with the cumulative number of reported cases in each jurisdiction

Jurisdiction	Reported Cases[a]	Estimated Infections[b]	Estimated Infections, Lower[b]	Estimated Infections, Upper[b]	Median Collection Date[c]	Ratio of Estimated Infections to Reported Cases	Ratio Range
AK	5,363	2,954	812	6,056	16-Sep	0.6	0.2-1.1
AL	122,183	423,227	301,124	550,195	11-Sep	3.5	2.5-4.5
AR	59,579	188,412	137,571	240,151	11-Sep	3.2	2.3-4.0
AZ	203,953	375,121	213,958	591,858	17-Sep	1.8	1.0-2.9
CA	697,085	1,918,289	1,241,016	2,681,690	11-Sep	2.8	1.8-3.8
CO	56,771	182,528	105,092	272,132	11-Sep	3.2	1.9-4.8
CT	52,495	111,027	73,779	156,512	11-Sep	2.1	1.4-3.0
DC	14,077	44,492	30,597	56,745	16-Sep	3.2	2.2-4.0
DE	17,429	71,212	46,430	99,697	14-Sep	4.1	2.7-5.7
FL	615,805	1,750,842	1,349,178	2,199,881	11-Sep	2.8	2.2-3.6
GA	265,333	1,338,673	1,082,266	1,625,973	11-Sep	5.0	4.1-6.1
HI	8,139	11,376	0	31,000	12-Sep	1.4	0.0-3.8
IA	66,135	238,070	174,167	313,876	16-Sep	3.6	2.6-4.7
ID	32,366	87,766	46,246	142,789	15-Sep	2.7	1.4-4.4
IL	229,482	576,967	397,466	778,265	11-Sep	2.5	1.7-3.4
IN	99,167	265,497	95,579	583,430	17-Sep	2.7	1.0-5.9
KS	41,537	101,807	67,193	140,785	11-Sep	2.5	1.6-3.4
KY	46,751	159,847	103,457	231,335	11-Sep	3.4	2.2-4.9
LA	148,169	582,952	467,294	727,990	14-Sep	3.9	3.2-4.9
MA	127,584	252,717	152,313	352,438	11-Sep	2.0	1.2-2.8
MD	109,319	612,350	474,872	761,236	16-Sep	5.6	4.3-7.0
ME	4,567	6,664	0	11,595	16-Sep	1.5	0.0-2.5
MI	113,025	368,427	252,920	490,904	14-Sep	3.3	2.2-4.3
MN	77,084	442,189	261,997	631,777	16-Sep	5.7	3.4-8.2
MO	80,958	213,152	130,936	305,112	11-Sep	2.6	1.6-3.8
MS	85,115	236,112	159,899	324,878	17-Sep	2.8	1.9-3.8
MT	7,691	22,918	6,980	45,628	16-Sep	3.0	0.9-5.9
NC	163,657	690,582	490,517	900,804	11-Sep	4.2	3.0-5.5
ND	12,000	9,026	0	25,575	15-Sep	0.8	0.0-2.1
NE	34,150	127,619	91,428	169,143	14-Sep	3.7	2.7-5.0
NH	7,216	9,405	3,493	17,736	11-Sep	1.3	0.5-2.5
NJ	192,972	1,341,159	1,123,553	1,565,869	17-Sep	7.0	5.8-8.1
NM	25,041	50,218	24,900	76,374	11-Sep	2.0	1.0-3.0
NV	67,841	227,982	172,448	291,993	11-Sep	3.4	2.5-4.3
NY	438,040	3,335,137	2,887,836	3,772,629	18-Sep	7.6	6.6-8.6

© 2020 Bajema KL et al. *JAMA Internal Medicine.*

OH	124,610	325,973	196,748	484,302	15-Sep	2.6	1.6-3.9
OK	56,258	195,907	134,784	256,246	11-Sep	3.5	2.4-4.6
OR	26,052	106,131	61,637	165,727	11-Sep	4.1	2.4-6.4
PA	136,771	1,419,821	1,132,020	1,753,671	17-Sep	10.4	8.3-12.8
PR[d]	.	101,608	60,288	144,622	.	.	.
RI	21,197	28,528	12,151	51,880	18-Sep	1.3	0.6-2.4
SC	115,951	386,562	276,541	505,009	11-Sep	3.3	2.4-4.4
SD	13,506	15,557	0	41,659	14-Sep	1.2	0.0-3.1
TN	150,812	445,623	329,894	569,998	11-Sep	3.0	2.2-3.8
TX	609,750	2,286,586	1,717,728	2,919,580	11-Sep	3.8	2.8-4.8
UT	52,115	155,313	100,192	240,583	14-Sep	3.0	1.9-4.6
VA	121,606	269,241	152,289	431,627	15-Sep	2.2	1.3-3.5
VT	1,637	10,625	1,687	25,937	17-Sep	6.5	1.0-15.8
WA	73,294	182,358	94,097	279,373	11-Sep	2.5	1.3-3.8
WI	77,127	219,579	127,125	331,102	16-Sep	2.8	1.6-4.3
WV	10,640	23,778	9,328	39,873	16-Sep	2.2	0.9-3.7
WY	4,031	8,728	0	23,099	21-Sep	2.2	0.0-5.7

[a]Cumulative cases reported on USAFacts[11] as of 14 days before the median collection date of commercial laboratory residual sera specimens in each jurisdiction.
[b]Estimated infections are calculated by multiplying the overall seroprevalence estimate in each jurisdiction by the underlying population using 2018 American Community Survey 5-year population totals.[1] Lower and upper estimated infections are calculated by multiplying the respective 95% confidence intervals for seroprevalence estimates by the underlying population.
[c]Median collection date of all samples in the corresponding jurisdiction during period 4.
[d]Cumulative cases not reported on USAFacts.[11]

© 2020 Bajema KL et al. *JAMA Internal Medicine.*

eTable 3. Overall, sex-, age-, and metropolitan/nonmetropolitan-stratified SARS-CoV-2 prevalence estimates and 95% confidence intervals by jurisdiction during period 1

			Overall		Male		Female		Age <18		Age 18-49		Age 50-64		Age ≥65		Metro Counties		Non-metro Counties	
Jurisdiction	Collection Dates	Number of Specimens	%	C.I.	%	C.I.	%	C.I.	%	C.I.	%	C.I.	%	C.I.	%	C.I.	%	C.I.	%	C.I.
AK	08/06 - 08/11/2020	242	0.3	0.00-1.12*	0.0	0.00-4.11**	0.7	0.00-2.35*	†		0.7	0.00-2.23*	0.0	0.00-4.35**	†		0.5	0.00-1.55*	†	
AL	07/29 - 08/13/2020	938	5.8	4.16-7.71	4.9	2.50-7.97	6.6	4.39-9.15	10.2	5.61-14.83	4.7	1.86-7.80	5.9	2.71-10.00	2.3	0.60-4.63	5.2	3.42-7.46	7.5	4.01-11.85
AR	07/29 - 08/13/2020	937	4.1	2.74-5.63	3.8	1.86-6.10	4.3	2.40-6.68	5.0	2.00-8.60	4.7	2.17-7.88	4.2	1.38-7.50	1.5	0.00-3.29	3.8	2.28-5.53	4.5	2.44-7.26
AZ	07/31 - 08/11/2020	591	8.2	4.11-13.59	9.0	2.20-17.70	7.5	3.26-13.56	†		10.8	3.61-20.32	7.1	0.74-14.83*	5.5	0.18-12.19*	8.6	4.19-14.20	†	
CA	07/30 - 08/05/2020	980	5.7	4.05-7.77	5.1	2.74-7.83	6.3	3.97-9.19	6.2	3.12-10.09	7.0	3.90-10.30	5.2	2.46-8.82	1.1	0.00-2.61*	5.7	4.05-7.82	†	
CO	07/30 - 08/07/2020	1,001	2.4	0.89-4.53	2.7	0.75-5.72	2.1	0.49-4.94	4.3	0.63-10.52*	1.1	0.00-2.75*	4.3	0.34-11.31*	0.8	0.00-2.07*	1.6	0.71-2.62	†	
CT	07/30 - 08/03/2020	994	3.4	2.04-4.72	4.2	2.13-6.42	2.5	1.18-4.10	3.9	1.48-6.46	3.1	1.00-5.62	3.7	1.30-6.30	2.7	0.50-5.17	3.5	2.15-4.97	†	
DC	07/30 - 08/13/2020	717	3.9	2.18-5.76	3.3	1.27-5.93	4.4	2.06-7.53	†		1.4	0.00-3.09*	5.5	2.28-9.15	6.4	3.28-9.94	3.9	2.18-5.76	NA	
DE	07/29 - 08/13/2020	804	5.7	3.55-8.22	4.3	2.13-7.12	7.0	4.02-10.92	†		8.3	4.31-12.72	5.0	2.24-8.34	0.9	0.00-2.29*	5.7	3.55-8.22	NA	
FL	07/31 - 08/03/2020	980	4.3	2.77-5.86	5.2	2.67-7.95	3.5	1.78-5.34	3.8	1.57-6.35	5.4	2.28-8.78	3.7	1.22-6.46	3.3	0.96-6.22	4.1	2.74-5.66	†	
GA	08/02 - 08/11/2020	985	6.8	4.83-8.81	8.1	5.08-11.86	5.6	3.35-8.43	8.6	4.78-12.54	7.8	4.18-11.09	4.9	2.02-7.86	2.8	0.80-5.52	6.5	4.52-8.33	8.6	3.19-14.70
HI	08/03 - 08/11/2020	5	†		†		†		†		†		†		†		†		†	
IA	07/29 - 08/13/2020	811	8.6	5.98-11.34	4.6	1.85-7.40	12.5	8.54-17.01	†		11.4	7.12-16.11	6.5	2.92-9.93	2.5	0.44-5.12	11.7	7.76-15.82	4.1	1.98-6.34
ID	08/04 - 08/11/2020	246	4.5	1.80-7.99	7.0	2.04-14.12	2.1	0.16-5.31*	†		10.3	3.75-19.04	†		1.3	0.00-4.51*	6.7	2.69-11.94	†	
IL	07/29 - 08/10/2020	1,004	3.9	2.52-5.21	3.5	1.62-5.64	4.3	2.59-6.32	5.1	1.96-8.55	3.8	1.60-6.24	3.5	1.43-5.78	3.1	1.18-5.51	4.4	2.85-5.89	†	

© 2020 Bajema KL et al. *JAMA Internal Medicine.*

			Overall		Male		Female		Age <18		Age 18-49		Age 50-64		Age ≥65		Metro Counties		Non-metro Counties	
Jurisdiction	Collection Dates	Number of Specimens	%	C.I.	%	C.I.	%	C.I.	%	C.I.	%	C.I.	%	C.I.	%	C.I.	%	C.I.	%	C.I.
IN	07/31 - 08/11/2020	579	2.2	1.10-3.63	1.4	0.00-3.30*	3.0	1.16-5.46	†		3.3	0.97-6.16	4.4	1.08-9.53	0.0	0.00-2.56**	2.1	1.02-3.69	†	
KS	07/29 - 08/08/2020	962	1.6	0.70-2.80	1.4	0.28-2.84	1.8	0.44-3.46	1.6	0.00-3.74	1.9	0.33-4.26	1.5	0.00-3.36	0.9	0.00-2.51*	1.3	0.44-2.31	2.3	0.40-4.96
KY	07/30 - 08/13/2020	975	2.4	1.32-3.62	2.2	0.65-4.06	2.7	1.16-4.39	4.1	1.66-7.21	2.1	0.34-4.34	2.2	0.42-4.31	1.3	0.00-3.17*	3.3	1.56-5.29	1.1	0.17-2.30
LA	07/28 - 08/13/2020	1,002	9.6	6.88-12.29	8.9	5.10-13.35	10.2	6.91-13.74	11.7	6.99-17.33	10.6	6.64-15.17	9.9	4.06-18.63	2.7	0.73-5.06	10.3	7.73-12.78	†	
MA	07/30 - 08/10/2020	978	4.2	2.89-5.81	4.8	2.83-7.36	3.7	1.94-5.43	6.2	3.13-9.64	3.4	1.19-6.05	4.6	1.81-7.29	3.5	1.29-6.01	4.3	2.93-5.89	†	
MD	07/31 - 08/11/2020	783	9.7	7.26-12.87	11.5	6.57-16.74	8.1	5.18-11.83	†		11.6	7.13-15.96	5.7	3.04-8.61	2.8	0.93-5.07	9.7	7.26-12.87	† †	
ME	07/30 - 08/11/2020	598	0.5	0.00-1.16	1.0	0.00-2.37	0.0	0.00-1.01**	†		0.4	0.00-1.46*	1.2	0.00-3.28*	0.4	0.00-1.56*	0.8	0.00-1.96	0.0	0.00-2.00**
MI	07/30 - 08/11/2020	982	3.4	2.16-4.84	3.9	2.00-6.07	2.8	1.31-4.67	6.7	3.64-10.40	3.6	1.39-6.48	1.8	0.00-3.83	0.3	0.00-1.28*	3.7	2.26-5.38	1.9	0.00-4.45*
MN	07/29 - 08/13/2020	879	3.5	2.06-5.01	3.2	1.08-5.61	3.8	1.95-5.68	3.7	1.03-7.02	4.5	2.16-7.08	1.6	0.32-3.10	3.2	0.00-9.08*	4.1	2.46-5.80	†	
MO	07/28 - 08/10/2020	979	2.5	1.44-3.61	1.7	0.55-3.19	3.3	1.45-5.19	3.2	1.12-5.45	2.9	0.96-5.15	2.1	0.33-4.46	1.1	0.00-3.25*	3.0	1.62-4.43	1.1	0.00-2.90*
MS	07/30 - 08/13/2020	769	7.1	3.75-11.09	4.0	1.23-7.81	10.0	4.05-17.18	†		6.2	2.83-10.12	5.0	2.09-8.73	2.6	0.54-5.49*	5.8	2.99-9.17	8.2	2.63-14.50
MT	07/29 - 08/10/2020	199	0.5	0.00-1.67*	0.0	0.00-4.20**	0.9	0.00-3.37*	†		†		†		†		1.3	0.00-4.75*	0.0	0.00-3.16**
NC	07/29 - 08/10/2020	928	2.5	1.31-3.72	1.6	0.31-3.10	3.3	1.66-5.20	6.4	2.84-10.49	1.6	0.35-3.50*	0.9	0.00-2.33*	1.0	0.00-2.52*	2.2	1.06-3.47	3.5	1.08-6.46
ND	07/29 - 08/12/2020	121	7.3	1.30-14.28	†		†		†		†		†		†		0.5	0.00-2.16*	†	
NE	07/28 - 08/13/2020	954	7.4	5.25-9.75	8.2	4.62-12.06	6.6	4.12-9.34	10.2	4.83-16.49	8.1	4.41-12.18	7.6	3.40-12.51	0.8	0.00-2.91*	8.2	5.82-10.97	5.9	1.60-11.20
NH	07/30 - 08/11/2020	809	0.8	0.22-1.48	1.0	0.14-2.08	0.6	0.00-1.39*	†		1.0	0.00-2.42*	0.3	0.00-1.31*	1.7	0.25-3.72	1.2	0.35-2.35	0.0	0.00-2.49**
NJ	07/31 - 08/11/2020	871	14.8	12.23-17.57	15.3	11.04-20.10	14.3	10.82-17.84	22.3	16.60-28.90	14.2	9.29-19.31	12.0	7.58-16.93	9.5	5.05-13.96	14.8	12.23-17.57	NA	

© 2020 Bajema KL et al. *JAMA Internal Medicine.*

			Overall		Male		Female		Age <18		Age 18-49		Age 50-64		Age ≥65		Metro Counties		Non-metro Counties	
Jurisdiction	Collection Dates	Number of Specimens	%	C.I.	%	C.I.	%	C.I.	%	C.I.	%	C.I.	%	C.I.	%	C.I.	%	C.I.	%	C.I.
NM	07/29 - 08/13/2020	964	2.0	1.09-3.02	0.6	0.00-1.84*	3.3	1.84-5.11	3.1	0.70-6.30*	1.8	0.63-3.69	2.6	0.68-4.86	0.3	0.00-0.99*	2.4	1.26-3.67	1.2	0.00-2.77*
NV	07/30 - 08/02/2020	979	5.1	3.63-6.66	5.3	2.87-8.07	4.9	3.19-6.86	8.9	5.36-12.85	4.2	1.69-7.30	4.0	1.78-6.61	3.1	1.14-5.64	5.7	4.00-7.35	†	
NY	07/31 - 08/11/2020	846	23.3	20.07-26.32	21.6	16.81-27.10	24.9	20.81-29.17	26.7	20.64-33.36	29.7	22.63-36.55	17.8	12.68-23.16	7.9	4.18-11.64	25.1	21.59-28.32	0.0	0.00-3.62**
OH	07/29 - 08/11/2020	786	2.3	1.11-3.61	2.5	0.70-4.69	2.1	0.65-3.76	3.3	0.00-8.03*	1.3	0.15-2.71	3.6	0.78-6.97	1.5	0.28-3.07	2.9	1.39-4.52	0.0	0.00-2.84**
OK	07/28 - 08/04/2020	979	1.6	0.85-2.49	1.4	0.29-2.74	1.8	0.78-3.03	3.6	1.28-6.20	0.4	0.00-1.32*	1.8	0.00-4.14*	1.5	0.00-3.50*	1.6	0.82-2.62	1.5	0.25-2.98
OR	08/05 - 08/11/2020	658	2.3	1.03-3.84	2.3	0.36-5.19*	2.3	0.73-4.00	5.6	0.87-12.05*	2.2	0.54-4.03	††		0.6	0.00-1.67*	2.8	1.18-4.50	†	
PA	07/31 - 08/11/2020	575	10.2	5.71-17.21	9.5	6.33-13.61	11.0	3.26-22.51	†		12.8	8.91-17.84	8.5	3.76-14.00	1.2	0.00-3.34*	11.5	6.45-19.44	0.5	0.00-1.93*
PR	07/27 - 08/07/2020	984	1.1	0.43-1.80	1.4	0.39-2.56	0.8	0.12-1.69*	0.0	0.00-1.49**	0.4	0.00-1.35*	2.5	0.53-5.15*	2.2	0.52-4.99*	1.1	0.45-1.90	†	
RI	07/30 - 08/11/2020	684	3.0	1.23-5.51	2.7	0.99-4.64	3.4	0.65-7.70	†		2.3	0.45-4.65	4.1	1.14-7.37	1.7	0.00-3.69	3.0	1.23-5.51	NA	
SC	07/30 - 08/13/2020	840	8.1	5.79-10.69	9.4	5.62-13.51	6.9	4.08-9.88	11.6	4.48-20.14	9.2	5.45-13.32	6.9	3.74-10.92	2.1	0.52-4.25*	8.2	5.78-11.07	7.2	2.85-11.76
SD	07/29 - 08/12/2020	47	†		†		†		††		†		†		†		†		†	
TN	07/30 - 08/11/2020	1,003	6.3	4.41-8.25	6.4	3.43-9.84	6.2	4.22-8.66	8.3	4.93-12.27	8.4	4.69-12.40	2.5	0.69-5.21	2.6	0.66-5.01	7.4	5.20-9.64	2.7	0.32-6.40*
TX	07/29 - 08/05/2020	986	5.9	4.04-7.95	6.2	3.61-8.88	5.5	2.95-8.31	8.4	4.78-13.05	5.6	2.94-9.06	5.5	2.32-9.42	1.9	0.34-4.02*	5.9	4.07-8.15	5.3	1.02-11.70*
UT	07/30 - 08/11/2020	880	3.2	1.70-5.03	2.2	0.62-3.92	4.2	1.77-7.50	†		4.3	1.96-6.73	3.1	1.13-5.53	1.3	0.00-3.26*	3.3	1.71-5.18	1.9	0.00-6.76*
VA	07/31 - 08/11/2020	730	4.1	1.80-6.93	2.7	0.57-5.24	5.5	1.54-10.71	†		3.9	1.46-6.77	2.4	0.26-5.12	2.2	0.39-4.72	4.6	2.03-7.86	0.6	0.00-2.08*
VT	07/30 - 08/11/2020	346	0.5	0.00-1.84*	1.0	0.00-3.74*	0.0	0.00-1.67**	†		1.1	0.00-4.42*	0.0	0.00-3.77**	0.0	0.00-3.89**	1.3	0.00-5.28*	0.0	0.00-1.46**
WA	07/29 - 08/11/2020	684	2.1	0.81-3.70	2.0	0.25-4.27*	2.3	0.72-4.20	0.5	0.00-1.84*	3.9	1.27-7.07	1.0	0.00-2.34*	††		2.3	0.90-3.97	†	

© 2020 Bajema KL et al. *JAMA Internal Medicine.*

			Overall		Male		Female		Age <18		Age 18-49		Age 50-64		Age ≥65		Metro Counties		Non-metro Counties	
Jurisdiction	Collection Dates	Number of Specimens	%	C.I.	%	C.I.	%	C.I.	%	C.I.	%	C.I.	%	C.I.	%	C.I.	%	C.I.	%	C.I.
WI	07/30 - 08/13/2020	814	1.8	0.76-3.18	2.0	0.26-3.96	1.7	0.41-3.40	2.4	0.00-6.28*	2.2	0.45-4.31	1.4	0.00-3.65*	0.8	0.00-2.18*	2.1	0.76-3.69	1.2	0.00-3.29*
WV	07/30 - 08/13/2020	317	1.2	0.23-2.50	1.5	0.00-3.48*	0.9	0.00-2.55*	†		1.7	0.00-4.74*	0.4	0.00-1.85*	†		1.9	0.37-3.86	†	
WY	07/29 - 08/11/2020	71	†		†		†		†		†		†		†		†		†	

† † No specimens were collected for the subgroup in period 1. Estimates are not shown.
† Because of small cell size (n < 75) for the subgroup in period 1, estimates are not shown.
* The confidence interval surrounding the estimate is large (i.e., high variance) relative to the estimate itself (RHWCI > 1.0). Associated point estimates should be interpreted with caution.
** No positive specimens were reported for the subgroup in period 1; confidence intervals were computed separately using Clopper-Pearson Exact method.
*** No specimen records received and analyzed for the jurisdiction in period 1.
NA indicates jurisdictions that do not have non-metro counties.

© 2020 Bajema KL et al. *JAMA Internal Medicine.*

eTable 4. Overall, sex-, age-, and metropolitan/nonmetropolitan-stratified SARS-CoV-2 prevalence estimates and 95% confidence intervals by jurisdiction during period 2

			Overall		Male		Female		Age <18		Age 18-49		Age 50-64		Age ≥65		Metro Counties		Non-metro Counties	
Jurisdiction	Collection Dates	Number of Specimens	%	C.I.	%	C.I.	%	C.I.	%	C.I.	%	C.I.	%	C.I.	%	C.I.	%	C.I.	%	C.I.
AK	08/12 - 08/26/2020	704	1.3	0.50-2.33	0.9	0.00-2.20*	1.7	0.53-3.20	†		2.5	0.81-4.85	0.8	0.00-2.65*	0.0	0.00-2.32**	1.4	0.56-2.53	0.9	0.00-2.70*
AL	08/12 - 08/26/2020	974	7.6	5.33-9.76	8.8	5.22-12.38	6.5	4.06-9.28	11.0	6.16-16.57	9.9	6.01-14.37	3.6	1.46-6.12	2.0	0.35-4.16*	7.3	4.88-9.67	8.5	4.47-13.30
AR	08/11 - 08/25/2020	998	3.0	1.76-4.35	3.3	1.26-5.70	2.8	1.33-4.36	3.9	0.84-7.45	3.2	1.08-5.60	3.9	1.44-7.18	0.5	0.00-1.50*	3.8	2.06-5.48	1.7	0.49-3.20
AZ	08/12 - 08/26/2020	1,304	4.7	2.70-7.61	5.5	1.82-10.69	3.9	1.82-7.01	†		7.4	3.94-12.04	5.6	0.66-12.71*	0.0	0.00-3.36**	5.0	2.85-8.01	†	
CA	08/13 - 08/19/2020	980	4.3	2.85-6.06	4.8	2.62-7.31	3.9	1.83-6.05	5.6	2.55-8.94	4.8	2.22-7.97	2.3	0.67-4.59	3.5	1.21-6.77	4.3	2.85-6.06	††	
CO	08/10 - 08/25/2020	1,019	3.3	1.83-4.80	3.4	1.34-5.69	3.2	1.61-5.11	3.3	1.18-5.69	4.8	2.05-7.81	1.7	0.34-3.33	0.5	0.00-1.65*	3.8	2.09-5.49	†	
CT	08/11 - 08/24/2020	983	2.4	1.45-3.52	2.2	0.85-3.87	2.6	1.38-4.26	5.1	1.95-8.16	1.0	0.00-2.70*	2.8	0.71-5.11	2.0	0.32-3.97	2.5	1.48-3.63	†	
DC	08/13 - 08/27/2020	781	6.8	4.59-9.21	5.5	2.48-9.17	8.0	4.85-11.64	†		8.3	4.77-12.02	5.5	2.52-8.91	2.9	1.05-5.23	6.8	4.59-9.21	NA	
DE	08/12 - 08/27/2020	1,004	8.5	5.79-11.25	9.7	5.32-14.62	7.3	4.14-10.62	†		9.3	5.26-13.82	6.3	3.29-9.23	2.1	0.68-3.72	8.5	5.79-11.25	NA	
FL	08/14 - 08/14/2020	978	4.5	3.15-6.06	4.6	2.41-7.31	4.3	2.42-6.34	6.4	3.13-9.94	5.4	2.47-8.88	2.7	0.75-5.38	2.3	0.44-4.78*	4.6	3.22-6.23	†	
GA	08/13 - 08/26/2020	1,031	7.4	5.52-9.51	8.3	5.35-11.68	6.5	4.23-9.32	9.6	5.64-13.96	6.4	3.38-9.84	7.2	3.45-11.88	6.8	2.80-12.09	8.1	6.03-10.22	†	
HI	08/14 - 08/26/2020	9	†		†		†		†		†		††		†		†		†	
IA	08/12 - 08/27/2020	980	9.4	7.12-11.63	7.8	5.02-11.66	10.9	7.54-14.88	†		11.9	7.93-15.81	9.1	6.01-12.49	4.5	1.92-7.39	12.0	8.19-15.40	5.7	3.55-8.13
ID	08/12 - 08/26/2020	651	4.6	1.67-9.05	4.8	0.69-12.76*	4.3	1.42-8.73	†		2.6	0.54-4.99	5.1	1.44-9.73	3.1	1.05-5.78	5.7	1.80-11.65	2.3	0.00-6.70*
IL	08/12 - 08/27/2020	985	4.9	3.40-6.65	5.1	3.01-7.38	4.8	2.44-7.08	3.5	1.17-6.40	7.4	4.23-10.90	3.4	1.30-6.12	2.0	0.58-3.71	5.6	3.84-7.52	†	

© 2020 Bajema KL et al. *JAMA Internal Medicine.*

			Overall		Male		Female		Age <18		Age 18-49		Age 50-64		Age ≥65		Metro Counties		Non-metro Counties	
Jurisdiction	Collection Dates	Number of Specimens	%	C.I.	%	C.I.	%	C.I.	%	C.I.	%	C.I.	%	C.I.	%	C.I.	%	C.I.	%	C.I.
IN	08/12 - 08/26/2020	906	3.1	1.53-4.76	2.9	0.45-6.11	3.2	1.69-5.18	†		5.0	2.63-7.71	0.9	0.00-3.07*	5.4	0.00-14.17*	2.4	1.23-3.76	5.4	0.00-12.20*
KS	08/11 - 08/25/2020	1,016	3.7	2.02-5.47	4.4	1.71-7.11	3.0	1.08-5.04	2.1	0.28-4.30	6.0	2.51-9.99	3.4	0.74-6.92	0.4	0.00-1.41*	1.4	0.48-2.45	8.5	4.05-13.53
KY	08/12 - 08/26/2020	1,126	3.1	2.05-4.49	4.1	2.26-6.08	2.1	0.89-3.81	4.2	1.70-6.94	3.5	1.47-6.03	2.8	1.07-4.85	1.0	0.00-2.58*	4.5	2.84-6.56	1.2	0.20-2.54
LA	08/12 - 08/25/2020	989	11.8	9.27-14.81	10.4	7.00-14.49	13.2	9.31-17.57	12.2	7.68-17.67	11.0	6.84-16.36	16.7	10.74-23.60	6.8	3.46-11.33	12.3	9.96-15.14	†	
MA	08/12 - 08/27/2020	980	3.7	2.27-5.21	4.4	2.22-6.78	3.0	1.49-4.97	4.2	1.69-7.37	4.6	2.14-7.84	2.4	0.48-4.70	2.1	0.34-4.47	3.7	2.27-5.21	† †	
MD	08/10 - 08/26/2020	1,174	7.0	4.92-9.33	7.9	4.22-11.37	6.2	3.78-8.98	†		7.9	5.30-11.00	4.6	2.33-7.26	3.8	1.88-6.10	7.1	5.03-9.45	†	
ME	08/12 - 08/27/2020	609	0.6	0.00-1.44*	0.0	0.00-1.53**	1.2	0.00-2.82*	†		1.4	0.00-3.63*	0.0	0.00-2.10**	0.2	0.00-1.02*	0.6	0.00-1.34	0.6	0.00-2.25*
MI	08/12 - 08/25/2020	983	4.2	2.88-5.76	4.8	2.62-7.37	3.5	1.80-5.52	6.0	3.09-9.18	3.9	1.39-6.81	3.9	1.47-7.17	2.7	0.79-4.86	4.6	3.10-6.37	2.3	0.00-5.94*
MN	08/11 - 08/27/2020	978	4.5	3.05-5.87	4.3	2.49-6.41	4.6	2.85-6.68	2.3	0.38-4.80	5.6	3.00-8.50	5.3	2.65-7.98	3.6	1.50-6.03	5.8	3.92-7.56	†	
MO	08/12 - 08/21/2020	1,021	3.5	2.23-5.06	2.8	1.11-5.15	4.1	2.24-6.23	3.7	1.30-6.63	4.3	1.85-7.45	3.1	1.26-5.34	1.6	0.00-3.77*	3.4	2.15-4.95	3.6	0.62-7.88
MS	08/12 - 08/27/2020	968	10.0	6.69-14.71	8.1	4.60-12.80	11.8	6.69-19.81	†		13.0	8.64-17.97	9.1	5.65-13.25	3.1	1.16-5.95	9.5	6.14-14.94	10.4	5.47-17.92
MT	08/12 - 08/24/2020	383	1.3	0.38-2.49	0.7	0.00-2.51*	1.9	0.40-3.93	†		1.7	0.00-4.55*	0.0	0.00-2.78**	3.6	0.66-7.20	1.3	0.00-3.97*	1.3	0.21-2.88
NC	08/11 - 08/27/2020	982	3.8	2.33-5.29	3.2	1.24-5.68	4.3	2.33-6.43	4.1	1.72-6.93	3.4	1.27-6.11	6.1	3.20-9.98	1.3	0.25-3.12*	3.1	1.82-4.71	6.1	2.86-9.74
ND	08/12 - 08/26/2020	135	0.6	0.00-1.45*	†		1.3	0.00-2.99*	†		†		†		†		1.2	0.00-2.92*	†	
NE	08/11 - 08/25/2020	990	7.9	5.51-10.61	8.6	4.80-13.19	7.2	4.46-10.44	†		10.0	6.45-13.73	4.5	2.44-6.93	2.9	0.93-5.54	8.8	6.24-11.77	6.2	1.44-12.43
NH	08/13 - 08/25/2020	1,125	0.8	0.30-1.22	0.9	0.23-1.70	0.6	0.10-1.30	†		0.8	0.00-1.64	1.3	0.23-2.42	1.0	0.00-2.29	1.2	0.48-1.94	0.0	0.00-1.79**
NJ	08/10 - 08/26/2020	1,127	12.2	10.15-14.53	11.0	7.88-14.46	13.4	10.59-16.23	16.9	11.31-21.99	14.2	10.30-18.50	7.3	4.33-10.26	6.7	3.93-10.08	12.2	10.15-14.53	NA	

© 2020 Bajema KL et al. *JAMA Internal Medicine.*

			Overall		Male		Female		Age <18		Age 18-49		Age 50-64		Age ≥65		Metro Counties		Non-metro Counties	
Jurisdiction	Collection Dates	Number of Specimens	%	C.I.	%	C.I.	%	C.I.	%	C.I.	%	C.I.	%	C.I.	%	C.I.	%	C.I.	%	C.I.
NM	08/10 - 08/27/2020	976	2.5	1.47-3.79	2.2	0.50-4.15	2.8	1.39-4.47	4.1	0.98-8.07	2.2	0.55-4.28	2.7	0.94-5.16	0.8	0.00-2.00*	2.7	1.35-4.07	2.1	0.28-4.49*
NV	08/12 - 08/26/2020	972	7.9	6.13-9.83	8.9	5.93-12.39	7.0	4.83-9.36	9.7	5.94-13.45	7.8	4.68-11.11	8.0	4.71-11.44	5.4	2.80-8.70	8.7	6.76-10.84	†	
NY	08/10 - 08/26/2020	1,122	20.6	18.04-23.14	20.6	16.69-24.78	20.5	17.21-24.14	25.2	19.71-30.91	22.2	17.52-27.19	16.8	12.74-21.44	14.6	10.95-18.43	22.1	19.40-24.89	†	
OH	08/12 - 08/27/2020	985	2.1	1.06-3.29	1.7	0.37-3.50	2.4	1.04-4.22	2.5	0.00-6.35*	2.0	0.43-4.09	2.6	0.89-4.67	1.0	0.00-2.58*	2.3	1.13-3.82	1.0	0.00-2.31*
OK	08/10 - 08/18/2020	979	4.0	2.72-5.51	3.5	1.50-5.76	4.6	2.85-6.79	6.9	3.69-10.78	3.4	1.34-6.08	1.7	0.29-3.73*	3.8	1.23-6.57	4.0	2.55-5.80	4.0	1.96-6.56
OR	08/10 - 08/27/2020	1,086	2.4	1.41-3.60	1.5	0.42-2.85	3.2	1.53-5.18	2.0	0.00-5.11*	2.9	1.35-4.99	1.7	0.40-3.31	2.1	0.42-4.40	2.5	1.43-3.80	1.6	0.00-4.73*
PA	08/10 - 08/26/2020	887	10.1	7.53-13.74	15.5	10.51-22.76	4.8	2.84-7.24	†		15.7	12.87-18.78	9.5	4.73-14.73	2.1	0.00-4.87*	11.1	8.32-15.40	2.0	0.37-4.52*
PR	08/10 - 08/17/2020	986	2.2	1.18-3.26	1.6	0.38-3.31	2.6	1.11-4.54	1.0	0.00-2.45*	0.9	0.00-2.49*	3.2	1.06-5.93	5.0	1.25-10.63*	1.9	1.02-2.79	†	
RI	08/12 - 08/27/2020	570	3.6	1.69-5.89	3.5	0.82-6.73	3.8	0.84-7.53	†		1.7	0.00-3.72	3.3	0.78-6.51	2.5	0.00-5.72*	3.6	1.69-5.89	NA	
SC	08/12 - 08/27/2020	1,063	6.0	4.37-7.95	7.3	4.49-10.30	4.8	3.03-7.01	5.3	2.13-8.95	7.0	4.01-10.42	6.0	3.21-9.42	4.6	1.93-8.10	6.2	4.36-8.15	5.3	2.03-9.01
SD	08/12 - 08/26/2020	83	0.0	0.00-4.35**	†		†		†		†		†		†		†		†	
TN	08/12 - 08/26/2020	1,029	6.7	5.11-8.57	7.3	4.64-10.26	6.1	3.79-8.67	6.8	3.77-10.13	9.1	5.76-13.41	5.1	2.39-8.67	2.1	0.45-4.29*	7.7	5.63-9.85	3.4	1.19-6.61
TX	08/12 - 08/24/2020	1,001	6.5	4.69-8.47	6.1	3.36-9.23	6.9	4.52-9.76	8.7	5.17-12.89	6.4	3.31-10.14	5.3	2.39-9.23	3.7	1.47-6.51	6.5	4.54-8.39	†	
UT	08/15 - 08/25/2020	871	5.5	2.94-8.71	7.0	2.57-12.61	3.9	2.06-6.09	†		6.2	3.37-9.22	3.3	1.07-5.51	1.4	0.00-3.12	6.0	3.16-9.56	1.6	0.00-5.60*
VA	08/10 - 08/26/2020	1,243	4.7	2.51-7.22	1.1	0.21-2.31	8.1	3.99-12.57	†		3.3	1.96-5.06	2.9	0.83-5.32	1.1	0.16-2.37	5.2	2.84-8.13	0.8	0.00-2.02*
VT	08/13 - 08/27/2020	382	0.3	0.00-0.81*	0.0	0.00-2.51**	0.5	0.00-1.60*	†		0.0	0.00-2.24**	0.8	0.00-3.03*	0.5	0.00-1.79*	0.0	0.00-3.24**	0.4	0.00-1.25*
WA	08/12 - 08/27/2020	1,238	3.0	1.79-4.47	3.7	1.55-6.46	2.4	1.06-3.95	4.7	1.06-9.44	3.4	1.67-5.79	1.6	0.22-3.58*	1.2	0.13-2.77*	3.1	1.72-4.58	2.7	0.00-7.42*

© 2020 Bajema KL et al. *JAMA Internal Medicine.*

			Overall		Male		Female		Age <18		Age 18-49		Age 50-64		Age ≥65		Metro Counties		Non-metro Counties	
Jurisdiction	Collection Dates	Number of Specimens	%	C.I.	%	C.I.	%	C.I.	%	C.I.	%	C.I.	%	C.I.	%	C.I.	%	C.I.	%	C.I.
WI	08/12 - 08/27/2020	838	3.3	2.03-4.72	2.1	0.74-3.74	4.5	2.26-6.81	1.4	0.00-3.84*	4.3	1.92-7.18	3.8	1.50-6.22	2.2	0.00-5.29*	3.6	2.07-5.24	2.5	0.00-5.84*
WV	08/13 - 08/27/2020	637	2.2	0.52-4.90*	2.5	0.22-7.73*	1.9	0.30-4.49*	†		1.2	0.23-2.74*	0.8	0.00-2.73*	1.1	0.00-3.14*	3.6	0.85-7.72*	†	
WY	08/13 - 08/24/2020	86	0.8	0.00-3.11*	†		†		†		†		†		†		†		†	

† † No specimens were collected for the subgroup in period 2. Estimates are not shown.
† Because of small cell size (n < 75) for the subgroup in period 2, estimates are not shown.
* The confidence interval surrounding the estimate is large (i.e., high variance) relative to the estimate itself (RHWCI > 1.0). Associated point estimates should be interpreted with caution.
** No positive specimens were reported for the subgroup in period 2; confidence intervals were computed separately using Clopper-Pearson Exact method.
*** No specimen records received and analyzed for the jurisdiction in period 2.
NA indicates jurisdictions that do not have non-metro counties.

© 2020 Bajema KL et al. *JAMA Internal Medicine.*

eTable 5. Overall, sex-, age-, and metropolitan/nonmetropolitan-stratified SARS-CoV-2 prevalence estimates and 95% confidence intervals by jurisdiction during period 3

			Overall		Male		Female		Age <18		Age 18-49		Age 50-64		Age ≥65		Metro Counties		Non-metro Counties	
Jurisdiction	Collection Dates	Number of Specimens	%	C.I.	%	C.I.	%	C.I.	%	C.I.	%	C.I.	%	C.I.	%	C.I.	%	C.I.	%	C.I.
AK	08/26 - 09/09/2020	610	1.0	0.24-2.17*	1.0	0.00-3.19*	1.1	0.14-2.50*	†		1.6	0.00-3.78*	1.4	0.00-3.30*	0.5	0.00-1.86*	1.5	0.35-3.12*	0.1	0.00-1.21
AL	08/26 - 09/08/2020	955	9.9	7.19-12.79	7.7	4.19-12.18	11.9	8.24-16.73	†		11.6	7.67-16.19	7.0	3.59-10.48	3.4	1.64-5.60	9.3	6.61-12.49	11.7	4.40-18.43
AR	08/24 - 09/08/2020	992	4.9	3.50-6.41	4.3	2.36-6.62	5.6	3.54-7.74	7.8	3.61-11.73	3.6	1.69-5.91	4.3	1.72-7.34	5.1	2.23-8.24	5.6	3.79-7.66	3.8	1.97-5.58
AZ	08/26 - 09/09/2020	1,030	4.9	3.05-7.11	3.9	0.70-8.35	5.8	4.39-7.56	†		9.3	5.90-13.18	5.4	0.57-13.06*	†		5.1	3.15-7.48	†	
CA	08/28 - 09/09/2020	983	6.0	4.20-7.82	6.0	3.60-9.10	6.0	3.79-8.83	7.6	3.88-11.43	6.3	3.20-9.78	4.5	1.97-7.77	4.0	1.56-7.38	6.0	4.25-7.89	†	
CO	08/24 - 09/04/2020	1,025	3.6	2.31-5.09	4.4	2.14-7.06	2.8	1.52-4.25	4.0	1.83-6.38	3.6	1.24-6.44	3.1	1.09-5.24	3.4	1.08-5.82	4.1	2.64-5.82	†	
CT	08/26 - 09/04/2020	983	4.3	2.90-5.69	4.6	2.53-6.63	4.1	2.33-5.90	6.9	3.91-10.32	5.1	2.68-7.98	2.6	0.67-5.10	1.4	0.00-3.08	4.6	3.06-5.99	†	
DC	08/27 - 09/10/2020	643	5.0	2.83-7.55	5.7	2.47-10.11	4.3	1.98-7.19	†		5.7	2.64-9.24	4.9	1.52-9.31	3.3	0.94-6.48	5.0	2.83-7.55	NA	
DE	08/26 - 09/10/2020	914	4.5	2.42-7.12	3.9	1.13-7.88	5.0	2.29-8.77	†		5.3	2.73-8.69	2.9	0.80-5.42	1.1	0.23-2.28	4.5	2.42-7.12	NA	
FL	08/27 - 09/09/2020	981	5.7	3.93-7.49	6.0	3.12-9.09	5.3	3.37-7.70	3.8	1.11-6.51	9.3	5.38-13.53	5.1	2.22-8.97	0.7	0.00-1.87*	5.9	4.07-7.74	†	
GA	08/25 - 09/10/2020	1,020	8.7	6.69-11.14	8.2	5.51-11.58	9.2	6.52-12.51	13.4	8.84-18.51	6.8	3.83-10.60	9.3	5.30-13.99	5.5	2.58-9.08	9.1	7.02-11.61	6.7	1.99-12.62
HI	08/28 - 09/09/2020	12	†		†		†		†		†		†		†		†		†	
IA	08/25 - 09/10/2020	1,006	8.4	6.15-10.74	7.6	4.68-11.00	9.3	5.93-12.85	†		9.8	6.72-12.99	8.7	5.66-12.16	3.5	1.20-6.02	10.1	6.52-13.73	5.9	3.68-8.30
ID	08/26 - 09/10/2020	790	5.7	2.90-9.70	8.0	2.67-15.94	3.4	1.86-5.61	†		7.0	3.23-12.14	4.4	1.56-8.13	3.4	0.88-6.90	7.6	3.60-13.51	1.8	0.00-5.93*
IL	08/26 - 09/03/2020	979	5.6	3.90-7.67	5.0	3.04-7.34	6.1	3.50-9.83	9.6	5.89-13.41	3.1	1.28-5.33	6.7	1.86-14.30	4.9	2.30-7.94	5.6	4.05-7.24	†	

© 2020 Bajema KL et al. *JAMA Internal Medicine.*

			Overall		Male		Female		Age <18		Age 18-49		Age 50-64		Age ≥65		Metro Counties		Non-metro Counties	
Jurisdiction	Collection Dates	Number of Specimens	%	C.I.	%	C.I.	%	C.I.	%	C.I.	%	C.I.	%	C.I.	%	C.I.	%	C.I.	%	C.I.
IN	08/26 - 09/09/2020	984	2.4	1.36-3.52	2.0	0.36-4.09	2.8	1.47-4.46	†		3.8	1.81-5.97	3.0	0.63-6.29	1.7	0.16-3.93*	2.6	1.46-3.91	†	
KS	08/24 - 09/03/2020	1,025	2.9	1.57-4.31	1.9	0.61-3.44	3.9	1.90-6.39	1.9	0.57-3.62	3.4	0.92-6.49	3.9	1.51-6.77	1.9	0.29-4.68	2.6	1.59-3.94	3.3	0.45-6.62
KY	08/26 - 09/08/2020	983	3.1	1.91-4.33	2.6	1.03-4.48	3.5	1.97-5.23	2.3	0.45-4.50	3.5	1.40-5.84	4.0	1.52-6.54	2.0	0.30-3.95	3.9	2.38-5.67	1.9	0.44-3.70
LA	08/26 - 09/08/2020	1,001	8.6	6.65-11.30	6.3	3.49-9.56	10.8	7.40-15.35	8.3	4.25-14.50	9.9	6.04-14.73	7.9	4.55-12.36	6.5	3.35-11.00	10.1	7.91-12.49	†	
MA	08/27 - 09/05/2020	981	3.6	2.36-5.08	4.5	2.28-6.98	2.8	1.39-4.66	5.3	2.09-8.98	2.1	0.39-4.58	7.0	3.46-10.67	1.3	0.00-3.19*	3.6	2.36-5.08	† †	
MD	08/26 - 09/08/2020	980	7.9	5.72-10.35	9.9	6.23-14.31	6.0	3.52-9.00	15.6	7.84-24.49	6.2	3.54-9.16	7.7	4.70-11.14	1.4	0.00-3.31*	8.1	5.83-10.56	†	
ME	08/26 - 09/10/2020	759	1.5	0.44-3.22	1.5	0.43-2.78	1.6	0.00-4.96*	†		0.3	0.00-1.19*	3.2	1.05-6.49	0.3	0.00-1.36*	2.4	0.68-5.29	0.2	0.00-0.88*
MI	08/26 - 09/03/2020	976	3.4	2.16-4.95	3.6	1.57-5.86	3.2	1.74-4.89	5.2	2.25-8.28	3.3	1.09-6.51	2.9	1.00-5.36	1.9	0.34-3.81	3.8	2.40-5.44	1.4	0.00-4.59*
MN	08/26 - 09/08/2020	974	9.2	6.71-11.95	6.9	4.05-10.79	11.5	7.97-15.79	4.7	0.51-11.63*	11.8	7.88-17.03	10.6	5.42-17.43	7.4	3.71-12.79	8.8	6.81-11.07	†	
MO	08/24 - 09/10/2020	1,024	2.9	1.74-4.17	2.3	0.79-3.95	3.5	1.88-5.22	5.8	3.02-8.96	2.7	0.85-5.12	2.0	0.33-3.86	0.6	0.00-1.70*	3.2	1.92-4.70	2.2	0.00-4.49
MS	08/26 - 09/10/2020	987	8.4	4.89-12.73	4.8	2.17-8.23	11.8	5.49-20.00	†		9.9	5.73-14.11	4.0	1.70-7.26	6.4	2.79-11.09	6.6	3.56-10.73	9.9	4.23-18.04
MT	08/24 - 09/09/2020	518	0.9	0.23-1.82	1.0	0.00-2.33*	0.9	0.00-1.97	†		1.2	0.00-2.93*	0.4	0.00-1.42*	2.2	0.00-5.10	2.0	0.32-4.33	0.4	0.00-1.06*
NC	08/26 - 09/09/2020	982	3.8	2.33-5.26	2.8	1.31-4.65	4.7	2.57-7.26	6.5	3.20-9.94	2.9	0.96-5.52	3.1	0.98-5.53	3.0	0.91-5.74	3.9	2.40-5.53	3.3	0.96-6.91
ND	08/26 - 09/09/2020	109	0.2	0.00-0.99*	†		0.5	0.00-2.04*	†		†		†		†		0.5	0.00-1.99*	†	
NE	08/24 - 09/10/2020	938	6.3	4.47-8.27	5.1	2.85-7.73	7.4	4.26-10.72	9.8	4.14-15.92	6.2	3.48-9.55	5.6	3.26-8.54	1.6	0.29-3.08	8.8	6.28-11.71	1.5	0.00-3.54*
NH	08/26 - 09/03/2020	1,147	1.6	0.73-2.84	1.6	0.19-3.90*	1.7	0.76-2.86	†		1.8	0.59-3.33	1.2	0.38-2.27	1.0	0.00-2.22*	2.3	0.99-4.16	0.5	0.00-1.54*
NJ	08/26 - 09/07/2020	944	12.8	10.49-15.35	11.6	8.27-15.28	14.0	10.83-17.51	17.4	12.23-22.94	12.2	8.35-16.66	14.5	9.89-19.19	5.4	2.34-9.10	12.8	10.49-15.35	NA	

© 2020 Bajema KL et al. *JAMA Internal Medicine.*

			Overall		Male		Female		Age <18		Age 18-49		Age 50-64		Age ≥65		Metro Counties		Non-metro Counties	
Jurisdiction	Collection Dates	Number of Specimens	%	C.I.	%	C.I.	%	C.I.	%	C.I.	%	C.I.	%	C.I.	%	C.I.	%	C.I.	%	C.I.
NM	08/25 - 09/08/2020	985	3.7	2.28-5.55	3.2	1.11-6.06	4.1	2.27-6.36	2.0	0.00-4.97*	5.8	3.00-9.35	2.5	0.88-4.59	2.3	0.50-5.09*	4.1	2.46-6.06	2.9	0.60-5.80*
NV	08/27 - 08/29/2020	980	6.5	4.63-8.58	6.5	3.71-9.79	6.6	4.38-8.80	5.4	2.55-8.62	7.1	4.02-10.40	6.7	3.59-10.15	6.5	2.42-12.23	6.9	4.88-8.88	†	
NY	08/26 - 09/10/2020	1,043	19.5	16.88-22.38	18.7	14.92-22.86	20.3	16.36-24.53	18.2	14.05-23.29	23.0	17.51-29.15	20.2	15.04-25.20	10.8	7.22-14.96	20.7	17.89-23.78	4.0	0.00-9.67*
OH	08/27 - 09/10/2020	983	5.0	3.17-7.14	5.5	2.59-8.84	4.6	2.03-7.39	10.2	3.42-17.85	4.2	1.93-7.02	4.0	1.55-7.12	1.1	0.00-2.78*	4.6	2.74-6.71	6.8	1.81-13.43
OK	08/24 - 09/01/2020	979	5.4	3.83-7.29	5.5	2.88-8.23	5.4	3.16-7.82	9.5	5.15-14.26	4.9	2.18-8.13	3.8	1.72-6.52	2.1	0.00-4.53*	6.3	4.02-8.74	3.8	1.53-6.28
OR	08/26 - 09/10/2020	968	1.9	0.92-2.94	2.3	0.70-4.06	1.5	0.33-2.97	1.6	0.00-3.98*	2.6	0.77-5.03	1.1	0.00-3.16*	1.4	0.29-3.14*	1.8	0.83-2.96	2.2	0.00-5.85*
PA	08/26 - 09/09/2020	845	9.5	6.58-13.56	14.5	9.30-22.42	4.7	2.58-7.65	†		15.2	12.09-18.46	7.5	3.26-12.56	1.8	0.00-4.68*	10.7	7.34-15.17	0.6	0.00-2.67*
PR	08/24 - 09/03/2020	980	2.5	1.36-3.76	3.3	1.31-5.77	1.8	0.73-3.08	1.9	0.34-4.21*	3.3	1.13-6.01	0.4	0.00-1.44*	3.5	1.26-6.15	2.7	1.45-3.96	†	
RI	08/27 - 09/10/2020	786	3.1	1.73-4.64	3.5	1.34-6.23	2.6	1.07-4.33	†		3.5	1.39-5.84	3.9	1.44-6.85	1.8	0.35-4.10	3.1	1.73-4.64	NA	
SC	08/26 - 09/10/2020	927	7.2	5.12-9.57	6.5	3.53-9.76	7.8	5.25-11.28	6.7	2.02-12.97	10.1	5.97-14.42	4.8	2.25-7.90	3.3	1.10-6.13	7.2	4.73-9.95	7.0	3.21-11.48
SD	08/26 - 09/10/2020	97	0.7	0.00-2.47*	1.3	0.00-4.89*	†		††		†		†		†		†		†	
TN	08/25 - 09/08/2020	950	5.4	3.62-7.31	5.6	3.18-8.52	5.2	2.96-7.74	5.3	2.05-9.01	7.0	3.81-11.03	3.5	1.11-6.07	4.0	1.59-6.91	6.3	4.13-8.58	2.4	0.39-5.05*
TX	08/25 - 09/02/2020	979	5.8	4.04-7.92	6.8	4.08-10.31	4.9	2.63-7.54	6.3	2.92-10.13	6.1	3.00-9.69	5.8	2.66-9.90	3.8	1.61-6.90	6.3	4.43-8.49	1.9	0.00-4.75*
UT	08/24 - 09/08/2020	1,104	4.9	2.82-7.67	6.1	2.77-10.49	3.8	1.77-6.77	†		4.3	2.25-6.76	6.5	3.68-10.08	2.3	0.58-4.20	5.4	3.09-8.58	0.7	0.00-2.07*
VA	08/26 - 09/08/2020	1,007	3.5	1.97-5.26	3.2	1.09-5.79	3.8	1.57-6.63	†		4.9	2.28-7.84	3.2	1.04-5.62	1.8	0.31-3.59	3.3	1.77-5.31	4.9	0.00-11.46*
VT	08/26 - 09/10/2020	485	0.4	0.00-0.94*	0.4	0.00-1.39*	0.4	0.00-1.09*	†		0.0	0.00-1.86**	0.5	0.00-1.92*	1.5	0.00-4.37*	0.6	0.00-1.96*	0.3	0.00-0.85*
WA	08/25 - 09/10/2020	820	4.5	2.63-6.94	4.6	1.74-8.62	4.5	2.08-7.85	6.0	2.36-9.99	5.6	1.95-10.80	2.5	0.71-4.76	1.7	0.16-3.57*	4.5	2.44-7.05	†	

© 2020 Bajema KL et al. *JAMA Internal Medicine.*

			Overall		Male		Female		Age <18		Age 18-49		Age 50-64		Age ≥65		Metro Counties		Non-metro Counties	
Jurisdiction	Collection Dates	Number of Specimens	%	C.I.	%	C.I.	%	C.I.	%	C.I.	%	C.I.	%	C.I.	%	C.I.	%	C.I.	%	C.I.
WI	08/25 - 09/10/2020	976	2.6	1.48-3.95	2.2	0.84-3.85	3.0	1.30-4.93	0.8	0.00-3.10*	4.2	1.78-7.03	2.3	0.44-4.33	1.4	0.20-2.85	2.4	1.20-3.66	3.2	0.62-6.47
WV	08/26 - 09/10/2020	1,128	1.5	0.78-2.38	1.5	0.50-2.67	1.5	0.56-2.79	†		2.5	1.00-4.44	1.1	0.00-2.46	1.5	0.27-3.11	1.9	0.86-3.06	0.9	0.16-2.02
WY	08/24 - 09/09/2020	70	†		†		†		†		†		†		†		†		†	

† † No specimens were collected for the subgroup in period 3. Estimates are not shown.
† Because of small cell size (n < 75) for the subgroup in period 3, estimates are not shown.
* The confidence interval surrounding the estimate is large (i.e., high variance) relative to the estimate itself (RHWCI > 1.0). Associated point estimates should be interpreted with caution.
** No positive specimens were reported for the subgroup in period 3; confidence intervals were computed separately using Clopper-Pearson Exact method.
*** No specimen records received and analyzed for the jurisdiction in period 3.
NA indicates jurisdictions that do not have non-metro counties.

© 2020 Bajema KL et al. *JAMA Internal Medicine.*

eTable 6. Overall, sex-, age-, and metropolitan/nonmetropolitan-stratified SARS-CoV-2 prevalence estimates and 95% confidence intervals by jurisdiction during period 4

			Overall		Male		Female		Age <18		Age 18-49		Age 50-64		Age ≥65		Metro Counties		Non-metro Counties	
Jurisdiction	Collection Dates	Number of Specimens	%	C.I.	%	C.I.	%	C.I.	%	C.I.	%	C.I.	%	C.I.	%	C.I.	%	C.I.	%	C.I.
AK	09/09 - 09/18/2020	681	0.4	0.11-0.82	0.2	0.00-0.95*	0.6	0.12-1.23*	†		0.0	0.00-1.96**	2.1	0.56-4.25	0.0	0.00-1.58**	0.5	0.09-1.14*	0.2	0.00-0.62*
AL	09/09 - 09/18/2020	905	8.7	6.19-11.31	7.4	3.95-11.21	9.9	6.44-13.76	16.1	8.47-24.39	8.1	4.76-11.73	4.6	2.12-7.92	4.9	2.29-7.99	7.3	4.94-10.16	13.2	7.46-19.71
AR	09/09 - 09/18/2020	990	6.3	4.60-8.03	6.8	4.22-9.26	5.7	3.75-8.02	8.6	4.20-13.10	6.9	3.82-9.94	5.2	2.49-8.54	2.6	0.64-4.99	5.9	3.77-8.35	6.9	4.35-9.62
AZ	09/09 - 09/23/2020	1,208	5.4	3.08-8.52	2.8	0.00-6.62*	8.0	4.34-12.86	†		9.6	5.03-15.86	4.4	1.07-9.09	0.1	0.00-0.42*	4.6	2.94-6.88	†	
CA	09/10 - 09/16/2020	979	4.9	3.17-6.85	5.9	3.22-8.67	4.0	2.03-6.21	8.1	4.35-12.01	4.7	1.83-8.13	3.6	1.18-6.65	2.1	0.38-4.66*	5.0	3.20-6.97	†	
CO	09/09 - 09/18/2020	1,032	3.3	1.90-4.92	2.7	0.91-5.05	3.9	1.76-6.53	4.5	1.30-9.81	3.6	1.33-6.34	1.9	0.51-3.45	2.3	0.65-4.23	3.3	1.95-4.79	†	
CT	09/09 - 09/14/2020	982	3.1	2.06-4.37	3.2	1.76-4.96	3.0	1.59-4.61	4.1	1.50-7.20	2.0	0.52-3.86	4.3	1.74-7.27	3.1	0.83-5.63	2.9	1.86-4.04	†	
DC	09/08 - 09/24/2020	976	6.5	4.47-8.29	7.1	4.36-9.97	5.9	3.49-8.57	7.5	2.70-12.52	6.0	3.31-9.02	8.7	4.43-12.74	4.1	1.82-6.63	6.5	4.47-8.29	NA	
DE	09/09 - 09/24/2020	980	7.5	4.89-10.50	7.6	3.93-11.89	7.4	4.09-11.24	†		8.6	4.62-12.73	2.9	0.93-5.20	3.2	1.38-5.60	7.5	4.89-10.50	NA	
FL	09/11 - 09/11/2020	979	8.5	6.55-10.68	9.3	6.12-12.83	7.8	5.27-10.68	13.0	8.08-18.74	10.0	6.19-13.81	6.5	3.37-10.60	3.0	1.04-5.76	8.8	6.78-10.99	†	
GA	09/09 - 09/18/2020	1,100	13.0	10.51-15.79	14.0	9.71-18.00	12.1	8.45-15.24	18.7	13.95-23.87	15.1	10.33-20.63	5.4	2.75-8.90	6.3	2.78-10.57	12.7	10.07-15.50	14.7	7.30-24.22
HI	09/08 - 09/22/2020	516	0.8	0.00-2.18*	1.4	0.00-3.97*	0.3	0.00-1.22*	†		1.9	0.00-5.10*	0.0	0.00-2.31**	0.3	0.00-1.77*	1.0	0.00-2.67*	†	
IA	09/09 - 09/24/2020	977	7.6	5.56-10.02	5.9	3.27-9.28	9.2	6.43-12.32	†		11.3	7.24-15.02	9.9	6.85-13.12	3.3	1.32-5.78	9.1	6.37-12.71	5.4	3.13-8.11
ID	09/08 - 09/18/2020	732	5.2	2.74-8.46	2.6	1.10-4.67	7.8	3.13-14.14	†		4.1	1.59-7.42	7.8	3.83-12.51	1.6	0.00-3.49*	5.9	2.51-10.32	3.8	1.07-8.35
IL	09/08 - 09/17/2020	992	4.5	3.10-6.07	6.2	3.88-8.91	2.9	1.67-4.44	5.7	2.87-8.71	3.5	1.33-6.13	6.9	3.73-10.43	2.6	0.72-5.17	5.1	3.50-6.86	†	

© 2020 Bajema KL et al. *JAMA Internal Medicine.*

			Overall		Male		Female		Age <18		Age 18-49		Age 50-64		Age ≥65		Metro Counties		Non-metro Counties	
Jurisdiction	Collection Dates	Number of Specimens	%	C.I.	%	C.I.	%	C.I.	%	C.I.	%	C.I.	%	C.I.	%	C.I.	%	C.I.	%	C.I.
IN	09/09 - 09/23/2020	1,155	4.0	1.44-8.79	5.5	0.72-15.45*	2.5	1.57-3.55	†		2.6	1.39-3.88	3.0	0.98-5.86	2.8	0.77-5.05	5.1	1.74-11.20	0.2	0.00-0.95*
KS	09/09 - 09/19/2020	1,007	3.5	2.31-4.84	3.4	1.77-5.58	3.5	1.82-5.36	5.0	2.04-8.01	3.6	1.67-6.05	2.3	0.39-4.48	2.3	0.45-4.48	3.7	2.36-5.12	3.0	0.83-5.44
KY	09/09 - 09/18/2020	980	3.6	2.33-5.21	5.0	2.67-7.52	2.4	1.12-4.02	4.1	1.14-7.60	4.8	2.22-7.63	2.5	0.62-4.74	1.5	0.00-3.42*	5.2	3.14-7.42	1.5	0.11-3.50*
LA	09/09 - 09/19/2020	952	12.5	10.02-15.61	11.8	8.05-16.83	13.1	9.57-16.89	15.1	9.78-21.76	15.5	10.87-21.50	8.4	4.52-12.90	4.8	1.91-8.22	12.3	9.59-15.52	13.6	8.06-20.82
MA	09/09 - 09/15/2020	988	3.7	2.23-5.16	3.1	1.28-5.55	4.2	2.30-6.44	5.3	2.31-8.46	5.2	2.27-8.41	1.4	0.00-3.23	0.6	0.00-1.95*	3.7	2.26-5.23	†	
MD	09/10 - 09/23/2020	1,242	10.2	7.91-12.68	12.7	9.66-15.94	7.8	4.76-11.01	11.5	5.26-18.50	13.5	9.92-17.33	6.4	4.06-8.95	3.6	1.81-5.81	10.3	8.10-12.99	†	
ME	09/08 - 09/24/2020	987	0.5	0.09-0.87	0.2	0.00-0.73*	0.7	0.15-1.41	†		0.8	0.00-1.66	0.4	0.00-1.10*	0.4	0.00-1.09*	0.8	0.16-1.47	0.0	0.00-1.35**
MI	09/08 - 09/22/2020	980	3.7	2.54-4.93	2.4	0.87-4.24	4.9	2.86-6.99	7.3	3.80-11.09	3.3	1.37-5.55	1.9	0.35-3.83	1.9	0.00-3.96	4.2	2.91-5.76	1.0	0.00-2.68*
MN	09/09 - 09/23/2020	1,033	8.0	4.74-11.43	8.5	3.90-13.28	7.5	3.69-12.69	10.6	1.68-21.27	8.7	4.27-14.25	6.4	3.18-10.90	4.0	1.76-6.66	6.5	4.67-8.64	†	
MO	09/08 - 09/24/2020	1,031	3.5	2.15-5.01	3.1	1.49-5.47	3.9	2.00-6.07	5.1	2.34-8.57	4.2	1.33-7.48	2.3	0.64-4.18	1.2	0.00-3.16*	3.5	2.14-5.08	3.8	0.55-7.67
MS	09/09 - 09/22/2020	980	7.9	5.35-10.87	7.6	4.42-11.42	8.2	4.35-12.81	†		11.9	6.94-17.26	7.6	3.81-11.71	6.2	2.84-10.21	7.7	4.67-11.32	8.0	4.23-12.46
MT	09/09 - 09/23/2020	508	2.2	0.67-4.38	2.0	0.31-4.49	2.5	0.33-6.41*	†		0.8	0.00-3.09*	3.3	0.73-7.47	2.3	0.00-5.39*	2.1	0.44-4.19	2.3	0.17-5.31*
NC	09/09 - 09/17/2020	925	6.8	4.83-8.87	6.9	3.93-10.07	6.7	4.38-9.36	10.1	5.68-16.04	8.4	4.58-12.59	4.4	1.76-7.37	0.4	0.00-1.39*	6.7	4.58-8.84	7.0	3.40-12.06
ND	09/09 - 09/24/2020	113	1.2	0.00-3.40*	†		2.4	0.00-6.98*	†		†		†		†		0.0	0.00-4.40**	†	
NE	09/09 - 09/18/2020	976	6.7	4.80-8.88	6.5	3.76-9.77	7.0	4.24-10.20	†		5.1	2.82-7.46	7.5	4.43-11.16	6.0	3.21-9.08	8.3	5.64-10.96	3.9	0.96-8.31
NH	09/09 - 09/17/2020	1,034	0.7	0.26-1.32	0.9	0.05-1.86	0.6	0.06-1.35	†		1.1	0.18-2.18	0.6	0.00-1.63*	1.1	0.00-2.67*	1.1	0.32-1.83	0.2	0.00-0.83*
NJ	09/09 - 09/23/2020	1,050	15.1	12.65-17.63	17.9	14.25-21.72	12.4	9.53-15.58	18.1	12.68-23.29	18.9	14.75-23.77	12.0	8.40-15.50	4.8	2.09-7.82	15.1	12.65-17.63	NA	

© 2020 Bajema KL et al. *JAMA Internal Medicine.*

			Overall		Male		Female		Age <18		Age 18-49		Age 50-64		Age ≥65		Metro Counties		Non-metro Counties	
Jurisdiction	Collection Dates	Number of Specimens	%	C.I.	%	C.I.	%	C.I.	%	C.I.	%	C.I.	%	C.I.	%	C.I.	%	C.I.	%	C.I.
NM	09/08 - 09/24/2020	1,014	2.4	1.19-3.65	2.1	0.63-4.23	2.6	1.30-4.35	4.0	1.13-8.35	1.7	0.42-3.73*	1.7	0.23-3.49*	2.4	0.45-5.13*	1.9	0.91-3.15	3.3	0.99-6.39
NV	09/09 - 09/12/2020	979	7.8	5.90-9.99	8.3	5.40-11.77	7.2	4.86-9.99	6.3	3.13-9.91	9.8	5.96-14.45	9.2	5.70-13.38	2.5	0.78-4.69	8.6	6.51-11.02	†	
NY	09/11 - 09/24/2020	1,168	17.0	14.72-19.23	16.0	12.51-19.44	18.0	14.96-21.24	21.4	16.82-26.39	17.2	13.15-21.68	16.5	12.10-20.83	11.2	7.66-14.59	18.3	15.83-20.69	†	
OH	09/10 - 09/22/2020	978	2.8	1.69-4.16	3.1	1.26-5.21	2.6	1.00-4.62	4.9	0.85-9.91	2.0	0.44-3.77	2.1	0.36-4.05	3.1	1.15-5.27	2.8	1.66-4.33	2.9	0.00-7.38*
OK	09/09 - 09/18/2020	980	5.0	3.44-6.54	3.7	1.68-6.04	6.2	3.93-8.52	7.1	3.68-10.91	5.5	2.77-8.59	3.8	1.44-6.85	1.6	0.00-3.65*	5.4	3.65-7.48	4.2	2.02-6.71
OR	09/08 - 09/22/2020	971	2.6	1.51-4.06	2.9	1.05-5.21	2.4	0.93-4.18	5.2	1.65-9.92	1.7	0.00-4.05*	2.1	0.70-4.09	2.5	0.52-4.97	2.6	1.37-3.93	3.0	0.00-7.81*
PA	09/09 - 09/23/2020	1,341	11.1	8.85-13.71	18.5	14.40-23.51	4.1	2.38-5.87	†		14.8	12.18-17.11	8.6	5.22-12.60	2.5	0.42-4.97	12.3	9.76-15.36	1.7	0.15-3.91*
PR	09/08 - 09/15/2020	970	3.0	1.78-4.27	3.9	2.00-6.30	2.1	0.82-3.70	2.6	0.50-5.34*	2.7	0.62-5.24	3.9	1.32-7.18	2.8	0.61-5.67	3.1	1.90-4.49	†	
RI	09/09 - 09/24/2020	795	2.7	1.15-4.91	3.9	1.05-8.15	1.5	0.54-2.69	†		1.7	0.32-3.77	4.1	1.73-6.76	1.6	0.00-3.31	2.7	1.15-4.91	NA	
SC	09/09 - 09/18/2020	975	7.8	5.58-10.19	8.9	5.65-12.93	6.8	4.20-9.92	9.6	3.20-17.53	10.4	6.69-14.54	3.9	1.60-6.46	3.9	1.68-6.34	8.1	5.64-10.87	5.9	2.62-10.38
SD	09/09 - 09/23/2020	99	1.8	0.00-4.82*	†		†		†		†		†		†		†		†	
TN	09/08 - 09/16/2020	1,028	6.7	4.96-8.57	7.4	4.67-10.56	6.0	3.87-8.39	10.8	7.19-15.29	6.8	3.50-10.78	5.8	3.27-9.01	1.7	0.33-3.57*	7.1	5.32-9.19	5.3	2.14-9.41
TX	09/09 - 09/24/2020	988	8.2	6.16-10.47	7.1	4.15-10.37	9.4	6.27-12.66	12.7	8.35-17.62	8.5	4.72-12.60	5.3	2.68-8.60	1.8	0.31-3.75*	9.2	6.86-11.50	†	
UT	09/09 - 09/18/2020	1,022	5.1	3.29-7.90	4.8	1.74-10.17	5.4	3.64-7.46	†		7.3	4.48-10.48	6.2	3.79-8.68	1.2	0.00-2.94*	5.7	3.58-8.79	0.3	0.00-1.07*
VA	09/10 - 09/23/2020	1,111	3.2	1.81-5.13	1.4	0.29-3.11	5.0	2.46-8.53	†		4.9	2.67-7.62	1.6	0.27-3.19	1.1	0.20-2.26	3.0	1.49-4.94	4.9	0.86-11.21*
VT	09/09 - 09/24/2020	497	1.7	0.27-4.15*	0.8	0.00-1.97*	2.7	0.00-7.64*	†		0.4	0.00-1.59*	0.6	0.00-2.24*	2.4	0.00-5.68*	0.4	0.00-1.82*	2.4	0.25-6.11*
WA	09/09 - 09/22/2020	968	2.5	1.29-3.83	2.9	1.00-5.55	2.0	0.84-3.57	4.2	0.88-8.66	2.2	0.51-4.46	1.1	0.00-2.68*	2.5	0.68-4.75	2.7	1.43-4.19	†	

© 2020 Bajema KL et al. *JAMA Internal Medicine.*

			Overall		Male		Female		Age <18		Age 18-49		Age 50-64		Age ≥65		Metro Counties		Non-metro Counties	
Jurisdiction	Collection Dates	Number of Specimens	%	C.I.	%	C.I.	%	C.I.	%	C.I.	%	C.I.	%	C.I.	%	C.I.	%	C.I.	%	C.I.
WI	09/09 - 09/24/2020	976	3.8	2.20-5.73	5.4	2.20-9.21	2.2	1.02-3.61	†		3.9	1.85-6.26	2.4	0.65-4.44	3.3	1.37-5.73	4.3	2.12-7.11	2.4	0.71-4.76
WV	09/09 - 09/22/2020	968	1.3	0.51-2.18	1.8	0.48-3.24	0.8	0.10-1.82	†		1.9	0.37-3.84	1.7	0.00-3.70	1.0	0.00-2.35*	0.5	0.08-1.08	2.5	0.82-4.68
WY	09/09 - 09/24/2020	111	1.5	0.00-3.97*	†		†		†		†		†		†		†		2.3	0.00-5.76*

† † No specimens were collected for the subgroup in period 4. Estimates are not shown.
† Because of small cell size (n < 75) for the subgroup in period 4, estimates are not shown.
* The confidence interval surrounding the estimate is large (i.e., high variance) relative to the estimate itself (RHWCI > 1.0). Associated point estimates should be interpreted with caution.
** No positive specimens were reported for the subgroup in period 4; confidence intervals were computed separately using Clopper-Pearson Exact method.
*** No specimen records received and analyzed for the jurisdiction in period 4.
NA indicates jurisdictions that do not have non-metro counties.

© 2020 Bajema KL et al. *JAMA Internal Medicine.*

Supplemental References

1. United State Census Bureau. American Community Survey Data. 2020; https://www.census.gov/programs-surveys/acs/data.html. Accessed October 7, 2020.
2. Baker R, Brick JM, Bates NA, et al. Report of the AAPOR Task Force on Non-Probability Sampling. 2013; https://www.aapor.org/AAPOR_Main/media/MainSiteFiles/NPS_TF_Report_Final_7_revised_FNL_6_22_13.pdf. Accessed October 21, 2020.
3. Iachan R, Deng Y. Weighting with propensity score matching: a national pilot survey using a mobile panel. Joint Statistical Meetings; 2017; Baltimore, Maryland.
4. Valliant R, Dever JA. Estimating Propensity Adjustments for Volunteer Web Surveys. 2011;40(1):105-137.
5. Terhanian G, Bremer J. A Smarter Way to Select Respondents for Surveys? 2012;54(6):751-780.
6. Battaglia MP, Hoaglin DC, Frankel MR. Practical Considerations in Raking Survey Data. *Survey Practice* 2009; https://www.surveypractice.org/article/2953-practical-considerations-in-raking-survey-data. Accessed October 21, 2020.
7. Iachan R. A new iterative method for weight trimming and raking. Joint Statistical Meetings; 2010; Vancouver, Canada.
8. Mercer A, Lau A, Kennedy C. For Weighting Online Opt-In Samples, What Matters Most? 2018; https://www.pewresearch.org/methods/2018/01/26/for-weighting-online-opt-in-samples-what-matters-most/. Accessed October 21, 2020.
9. Havers FP, Reed C, Lim T, et al. Seroprevalence of Antibodies to SARS-CoV-2 in 10 Sites in the United States, March 23-May 12, 2020. *JAMA Intern Med.* 2020; doi: 10.1001/jamainternmed.2020.4130.
10. Zhang F, Brick M, Kaufman S, Walter E. Variance estimation of imputed survey data. 1998; https://nces.ed.gov/pubs98/9814.pdf. Accessed October 21, 2020.
11. USAFacts. US Coronavirus Cases and Deaths. 2020; https://usafacts.org/visualizations/coronavirus-covid-19-spread-map. Accessed September 15, 2020.

© 2020 Bajema KL et al. *JAMA Internal Medicine.*
